# Exosomes secreted by *Fusobacterium nucleatum*-infected colon cancer cells transmit resistance to oxaliplatin and 5-FU by delivering hsa_circ_0004085

**DOI:** 10.1186/s12951-024-02331-9

**Published:** 2024-02-15

**Authors:** Bingqing Hui, Chenchen Zhou, Yetao Xu, Rui Wang, Yuwen Dong, Yirui Zhou, Jie Ding, Xiao Zhang, Jian Xu, Yanhong Gu

**Affiliations:** 1 Department of Oncology and Cancer Rehabilitation Center, The First Affiliated Hospital of Nanjing Medical University, Nanjing, Jiangsu China; 2 The First Clinical Medical College of Nanjing Medical University, Nanjing, Jiangsu China; 3 Department of Obstetrics and Gynecology, The First Affiliated Hospital of Nanjing Medical University, Nanjing, Jiangsu China; 4 Department of Oncology, The Second Affiliated Hospital of Nanjing Medical University, Nanjing, Jiangsu China; 5 Department of General Surgery, The Fourth Affiliated Hospital of Nanjing Medical University, Nanjing, Jiangsu China

**Keywords:** *Fusobacterium nucleatum*, Exosomes, hsa_circ_0004085, Chemoresistance of colon cancer, Endoplasmic reticulum stress

## Abstract

**Background:**

A large number of *Fusobacterium nucleatum* (Fn) are present in colorectal cancer (CRC) tissues of patients who relapse after chemotherapy, and Fn has been reported to promote oxaliplatin and 5-FU chemoresistance in CRC. Pathogens such as bacteria and parasites stimulate exosome production in tumor cells, and the regulatory mechanism of exosomal circRNA in the transmission of oxaliplatin and 5-FU chemotherapy resistance in Fn-infected CRC remains unclear.

**Methods:**

Hsa_circ_0004085 was screened by second-generation sequencing of CRC tissues. The correlation between hsa_circ_0004085 and patient clinical response to oxaliplatin/5-FU was analyzed. Exosome tracing experiments and live imaging systems were used to test the effect of Fn infection in CRC on the distribution of hsa_circ_0004085. Colony formation, ER tracking analysis and immunofluorescence were carried out to verify the regulatory effect of exosomes produced by Fn-infected CRC cells on chemotherapeutic resistance and ER stress. RNA pulldown, LC–MS/MS analysis and RIP were used to explore the regulatory mechanism of downstream target genes by hsa_circ_0004085.

**Results:**

First, we screened out hsa_circ_0004085 with abnormally high expression in CRC clinical samples infected with Fn and found that patients with high expression of hsa_circ_0004085 in plasma had a poor clinical response to oxaliplatin/5-FU. Subsequently, the circular structure of hsa_circ_0004085 was identified. Fn infection promoted hsa_circ_0004085 formation by hnRNP L and packaged hsa_circ_0004085 into exosomes by hnRNP A1. Exosomes produced by Fn-infected CRC cells transferred hsa_circ_0004085 between cells and delivered oxaliplatin/5-FU resistance to recipient cells by relieving ER stress. Hsa_circ_0004085 enhanced the stability of GRP78 mRNA by binding to RRBP1 and promoted the nuclear translocation of ATF6p50 to relieve ER stress.

**Conclusions:**

Plasma levels of hsa_circ_0004085 are increased in colon cancer patients with intracellular Fn and are associated with a poor response to oxaliplatin/5-FU. Fn infection promoted hsa_circ_0004085 formation by hnRNP L and packaged hsa_circ_0004085 into exosomes by hnRNP A1. Exosomes secreted by Fn-infected CRC cells deliver hsa_circ_0004085 between cells. Hsa_circ_0004085 relieves ER stress in recipient cells by regulating GRP78 and ATF6p50, thereby delivering resistance to oxaliplatin and 5-FU.

**Graphical Abstract:**

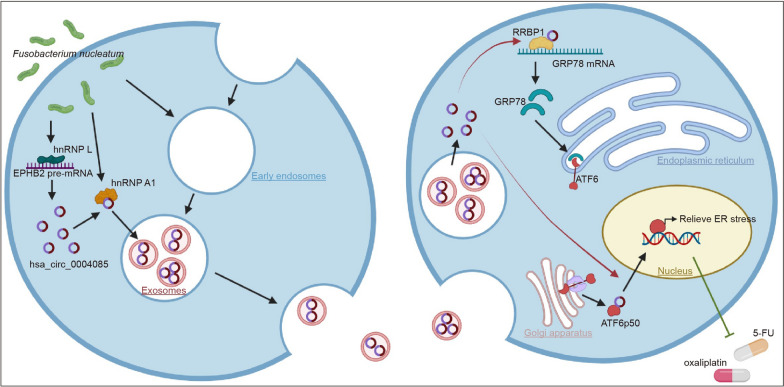

**Supplementary Information:**

The online version contains supplementary material available at 10.1186/s12951-024-02331-9.

## Background

Colorectal cancer (CRC) is the third most common cancer and the third leading cause of cancer-related death worldwide [1, 2]. Oxaliplatin and fluorouracil are the most commonly used baseline chemotherapeutic agents for CRC, among which oxaliplatin, a DNA synthesis inhibitor, causes DNA cross-link damage, blocks DNA replication and transcription and leads to cell death [3, 4]. Fluorouracil (also known as 5-fluorouracil, 5-FU), as well as capecitabine (which functions by converting into 5-FU in vivo), inhibits the synthesis of pyrimidine by inhibiting thymidylate synthase during DNA replication [5]. Most patients with metastatic colorectal cancer (mCRC) initially respond to XELOX (capecitabine combined with oxaliplatin) and FOLFOX regimens (mainly consisting of 5-FU and oxaliplatin). However, the disease progresses eventually due to drug resistance, and the 5-year survival rate of patients with mCRC is less than 10% [6]. Therefore, it is crucial to elucidate the mechanism of chemoresistance in colon cancer patients.

The gut microbiota plays a key role in the development and progression of CRC [7]. Mauro et al. observed that *Fusobacterium nucleatum* (*F. nucleatum*, Fn) was significantly increased in CRC tissues, and the amount of Fn was correlated with shorter survival [8–10]. Fn is an important component of the microbiota of the oral cavity, gastrointestinal tract, and upper respiratory tract and is an obligate anaerobic gram-negative bacterium that invades cells to obtain an anaerobic environment suitable for survival [11–13]. Studies have shown that Fn is abundant in CRC tissues from patients with recurrence after chemotherapy, and the results of bioinformatics and functional studies have shown that Fn promotes oxaliplatin and 5-FU chemoresistance in CRC [14].

Exosomes (exosomes, Ex) are nanosized extracellular biological vesicles that are excreted by most types of cells and circulate in body fluids [15]. Exosomes carry a variety of molecular and genetic components of the cells from which they are derived and influence cancer development and chemoresistance by delivering a variety of signals [16, 17]. During bacterial, parasitic, and fungal infections, pathogens not only secrete exosomes themselves but also stimulate tumor cells to produce exosomes to modulate the tumor microenvironment [18]. For instance, exosomal CagA derived from *Helicobacter pylori*-infected gastric epithelial cells induces macrophage foam cell formation and promotes atherosclerosis [19]. In addition, circRNAs are a class of newly discovered endogenous noncoding RNAs, and unlike conventional linear RNAs, the 3ʹ and 5ʹ ends of circRNAs are interconnected to form a covalently closed circle [20]. Exosomes are able to load and deliver circRNAs, and the circRNAs transferred between cells achieve drug resistance dissemination by interfering with some key pathways in internal and external factors of chemoresistance [21]. Exosomal circRNAs hold promise as targets for predicting chemoresistance as well as treatment resistance in CRC.

The present study screened abnormally highly expressed circRNAs in the tumor tissues and plasma of CRC patients infected with Fn. Fn was able to promote the secretion of exosomes from CRC cells and the enrichment of circRNAs in exosomes. We further found a correlation between circRNA levels and the patient's chemotherapeutic response to oxaliplatin or 5-FU and deeply explored the mechanism by which exosomal circRNA propagates oxaliplatin or 5-FU resistance.

## Materials and methods

### Fluorescence in situ hybridization (FISH)

The SABER probe targeting Fn designed by the ARB software package (http://www.arb-home.de) was used overnight hybridization with the eight CRC tissues at 42 °C. The next day, the tissue sections were blocked and subsequently incubated with mouse anti-digoxigen in-labeled horseradish peroxidase (anti-DIG-HRP). Fluorescein isothiocyanate-tricomycin A was added and reacted together at room temperature for 5 min. Finally, DAPI was used to stain the nuclei. Sections were observed under a Nikon upright fluorescence microscope, and images were collected. A Fluorescence In Situ Hybridization Kit (C10910, RIBOBIO) was used to detect hsa_circ_0004085 according to the operating instructions. The probe sequences are provided in Additional file 2: Table S1.

### Culture and infection of Fn

Fn (*F. nucleatum subsp. nucleatum*, CGMCC: 1.2526, original number: ATCC25586) was purchased from the China General Microbiological Culture Collection Center (CGMCC, Beijing, China). Fn were grown at 37 °C anaerobically on CDC anaerobic blood agar plates (Dijing Microbe, Beijing, China) for 72 h. Live Fn was heated at 100 ℃ for 10 min to obtain heat-inactivated (dead) Fn. *Escherichia coli* (CGMCC: 1.12252) was shaken overnight in LB medium at 220 rpm/min at 37 °C. The collected Fn was centrifuged in a centrifuge and resuspended in RPMI 1640. The Fn suspension was assessed spectrophotometrically at 595 nm and used for subsequent infection experiments. CRC cells were seeded in culture plates at a cell density of 1 × 10^6^ per well and infected with Fn at an MOI of 10:1 for 2 h. Fn in the medium was removed, and new medium was added.

### Extraction and detection of exosomes

CRC cells were cultured in normal medium until they were 80% confluent. Then the medium was replaced with exosome-depleted medium. Two days later, the conditioned medium was collected from each dish and subjected to differential centrifugation (15 min at 500 × g to remove cells, 30 min at 10,000 × g to remove cell debris and ultracentrifugation at 110,000 × g for 70 min) at 4 °C to collect pellet. The pellet was finally re-suspended in phosphate-buffered saline (PBS) and centrifuged at 110,000 × g for another 70 min to harvest exosomes without soluble and secreted proteins [22]. The protein content, used for the quantification of exosomes was measured using the Bicinchoninic acid (BCA) Protein Assay kit (Thermo Fisher, USA). Exosomes were processed by negative staining and imaged by transmission electron microscopy (TEM) (HT-7700, Hitachi). The exosome size and number were measured by a NanoSight NS300 system (Malvern Instruments Ltd., UK) equipped with nanoparticle tracking analysis (NTA) 3.0 Analysis software (Malvern Instruments Ltd., UK). For in vitro experiments, 10 µg exosomes were added to 1 × 10^5^ recipient cells. For in vivo experiments, 50 µg exosomes were injected intratumorally twice a week. GW4869 (D1692, Sigma Chemical Co, St. Louis, MO) was used for pharmacological depletion of exosomes, and ultracentrifugation was used for physical removal of exosomes from the culture medium.

### Tracer analysis of exosomes

CRC cells (1 × 10^5^) were incubated with 10 μg of exosomes stained with PKH26 (red) for 0 h, 2 h and 6 h. The cytoskeleton was stained with phalloidin labeled with green fluorescent dye at room temperature, and nuclei were then stained with DAPI. Finally, the exosomes and the cells were observed with laser confocal microscopy. To observe the migration path of hsa_circ_0004085, automatic circularized hsa_circ_0004085 vectors were constructed and labeled with Cy3 (orange-red). Exosomes isolated from CRC cells transfected with this vector were labeled with PKH67 (green) and then incubated with recipient cells. The localization of hsa_circ_0004085 and exosomes was observed with laser confocal microscopy.

### Endoplasmic reticulum tracking analysis

ER-Tracker Red (C1041, Beyotime, China) staining solution was used to stain the ER in living cells. After coincubation at 37 °C for 15–30 min, the ER-Tracker Red staining solution was removed, and the cells were washed with culture medium 1–2 times. Bright red fluorescence staining of the ER was observed under laser confocal microscopy.

### Statistical analysis

In this study, statistical significance was determined using GraphPad Prism software v.9.1.0. Quantitative data are expressed as the mean ± standard deviation, unless otherwise specified. The chi-square test (χ^2^ test) and Mann–Whitney U test were performed to analyze nonparametric variables. For parametric variables, Student’s t test was performed to analyze the significant differences between two groups, and analysis of variance (ANOVA) was applied to evaluate the differences between multiple groups. The Kaplan–Meier method was used to analyze overall survival.

Additional method information can be found in the Additional file 1.

## Results

### Hsa_circ_0004085 levels are abnormally elevated in tumor tissues and plasma of colon cancer patients infected with Fn

**Fig. 1:**
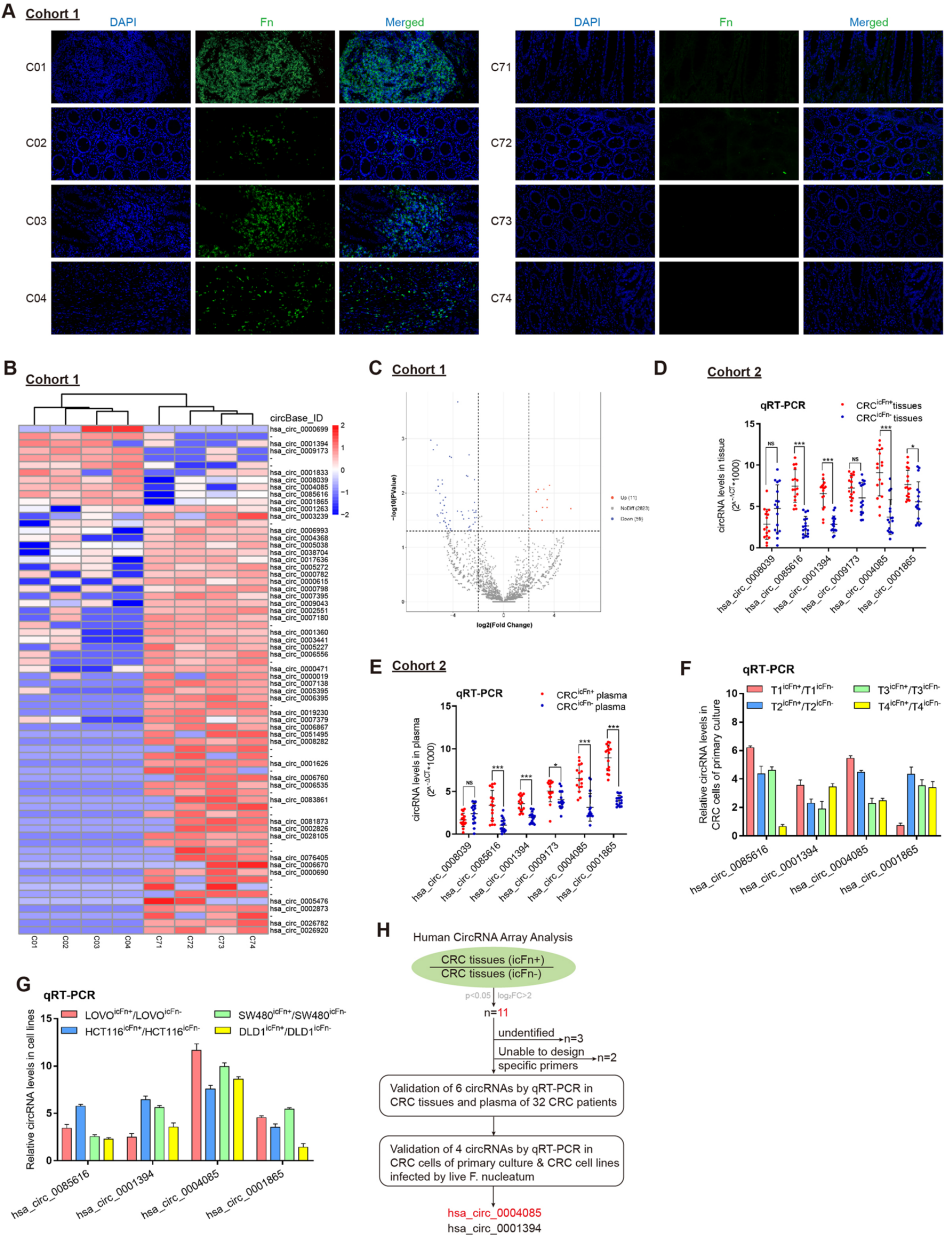
Hsa_circ_0004085 levels abnormally elevated in tumor tissues and plasma of CRC patients infected with Fn. **A** FISH was conducted in cohort 1 to identify four CRC^icFn+^ tissues (left) and four CRC^icFn−^ tissues (right). **B**, **C** Differential expression profiles of circRNAs between CRC^icFn+^ tissues and CRC^icFn−^ tissues were detected by next-generation sequencing, and 70 circRNAs with the most significant differences in expression levels are presented in a heatmap and volcano plot. **D**, **E** Expression levels of 6 circRNAs were measured by qRT-PCR in tumor tissues and plasma of 16 CRC^icFn+^ and 16 CRC^icFn−^ patients in cohort 2. **F**, **G** Expression levels of 4 circRNAs in CRC tumor primary cells and CRC cell lines infected with Fn were determined by qRT-PCR. **H** The circRNAs with abnormally high expression in the icFn+ group were selected by different tests at both the tissue and cellular levels. (*P < 0.05, **P < 0.01, ***P < 0.001, NS: not signifcant)

To identify circRNAs that might mediate the regulation of CRC by Fn infection, we performed three rounds of screening. As shown in Fig. 1A, fluorescence in situ hybridization (FISH) was first conducted in cohort 1 to identify intracellular Fn infection in colon cancer tissues (icFn ±). Differential expression profiles of circRNAs between the two groups were subsequently detected and analyzed by next-generation sequencing. As shown in Fig. 1B and C, heatmaps and volcano plots showed the 70 circRNAs that were significantly differentially expressed between the two groups (|log_2_FC|> 2, p < 0.05). We focused more on the 11 circRNAs that were significantly upregulated because highly expressed circRNAs have more potential to be clinical diagnostic markers. Excluding three circRNAs that were currently undefined and two for which specific primers could not be designed, we further detected the expression levels of the remaining six circRNAs by quantitative reverse transcription-polymerase chain reaction (qRT-PCR) in tumor tissues and plasma from patients in cohort 2 (16 CRC^icFn+^ patients and 16 CRC^icFn−^ patients). The results showed that four circRNAs still maintained a high expression trend in the icFn+ group relative to the icFn- group (Fig. 1D and E). As shown in Fig. 1F and G, in the third round of screening, living Fn was used to infect CRC tumor primary cells and CRC cell lines for 48 h, after which total RNA was extracted and these four circRNAs were detected. The results showed that only hsa_circ_0004085 and hsa_circ_0001394 were abnormally elevated in the icFn+ group in all the screening results (Fig. 1H). This subject only investigates the function of hsa_circ_0004085, while another study has been carried out by our group on hsa_circ_0001394.

To identify the origin of hsa_circ_0004085, we searched the CircInteractome database (https://circinteractome.irp.nia.nih.gov/), NCBI (https://www.ncbi.nlm.nih.gov/), and UCSC Genome Browser (http://genome.ucsc.edu/) and found that hsa_circ_0004085 was 750 bp in length and was generated from *EPHB2*. Sanger sequencing verified that hsa_circ_0004085 was composed of head-to-tail splicing of exons 2 and 3. PCR was performed to exclude head-to-tail splicing originating from transsplicing or genomic rearrangements (Additional file 1: Fig. S1A). To verify whether hsa_circ_0004085 has a covalent and closed loop structure, we treated cells with actinomycin D, an inhibitor of RNA synthesis that prevents the synthesis of RNA (especially mRNA), and found that hsa_circ_0004085 was more stable than GAPDH mRNA and EPHB2 mRNA (Additional file 1: Fig. S1B). Moreover, hsa_circ_0004085 was resistant to RNase R (an enzyme that degrades linear RNA but not circular RNA), whereas linear GAPDH mRNA and EPHB2 mRNA in the control group were not (Additional file 1: Fig. S1C). As shown in Additional file 1: Fig. S1D, hsa_circ_0004085 was mainly detected in non-mRNA samples (without a poly-A tail) rather than mRNA samples (with a poly-A tail).

### Hsa_circ_0004085 levels in the plasma of CRC patients correlate with chemotherapy response to oxaliplatin/5-FU

**Fig. 2:**
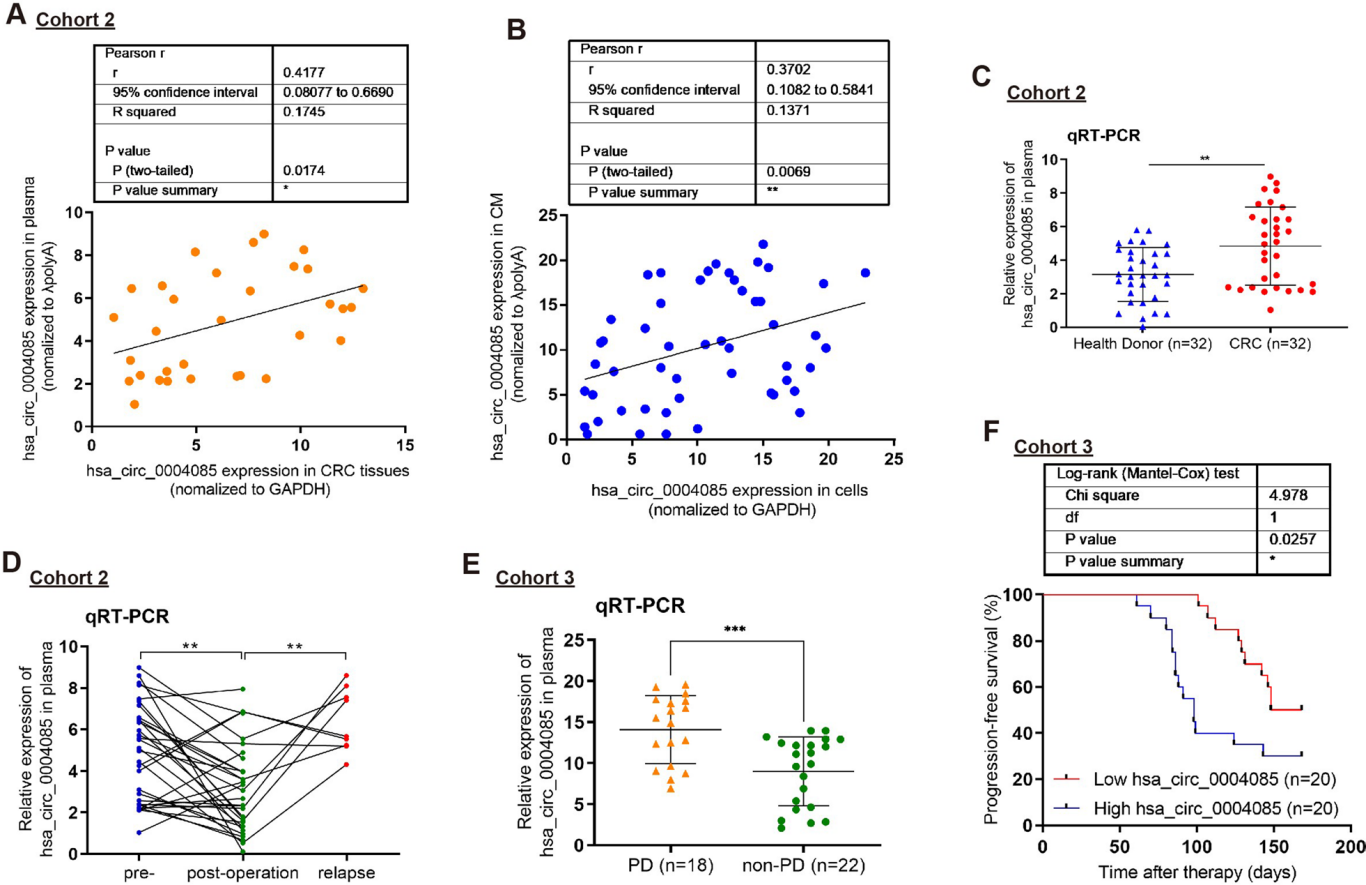
Hsa_circ_0004085 level in the plasma of CRC patients was correlated with the response to oxaliplatin/5-FU. **A** Hsa_circ_0004085 levels in tumor tissues and preoperative plasma of CRC patients were determined, and the correlation between them was analyzed. **B** Hsa_circ_0004085 levels in CRC cells and culture media were determined, and the correlation between them was analyzed. **C** Hsa_circ_0004085 levels in the preoperative plasma of CRC patients and in the plasma of healthy donors were measured by qRT-PCR. **D** Hsa_circ_0004085 levels in the plasma of CRC patients before surgery, after surgery, and after tumor recurrence were measured by qRT-PCR. **E** Hsa_circ_0004085 levels in the pretherapy plasma of CRC patients with different responses to chemotherapy were determined by qRT-PCR. **F** The median hsa_circ_0004085 level was used as the cut-off, and the effect of hsa_circ_0004085 in pretherapy plasma on PFS in CRC patients treated with XELOX or FOLFOX was analyzed using KM methods. (*P < 0.05, **P < 0.01, ***P < 0.001, NS: not signifcant)

To define the distribution characteristics of hsa_circ_0004085 in the intracellular and extracellular environments, we extracted and tested RNA from tumor tissues and plasma from CRC patients in cohort 2 or from CRC cells and cell culture medium (CM). As shown in Fig. 2A, there was a positive correlation between hsa_circ_0004085 levels in CRC tissues and those in plasma. Similar results were also obtained in cells and CM (Fig. 2B). Compared with healthy donors, hsa_circ_0004085 levels in plasma were elevated in CRC patients, decreased after tumor resection, and increased again after tumor recurrence (Fig. 2C, D), suggesting that plasma hsa_circ_0004085 is mainly produced by tumor cells.

To examine the potential relationship between hsa_circ_0004085 expression levels and chemotherapy response in CRC patients, before therapy, we examined the expression levels of hsa_circ_0004085 in the plasma of patients with CRC and for whom the first-line regimen was XELOX or FOLFOX based (cohort 3). The results showed that the expression levels of hsa_circ_0004085 were higher in the plasma of 18 patients with progressive disease (PD) during treatment than in 22 patients without PD (Fig. 2E). Therefore, abnormally high expression of hsa_circ_0004085 may potentially promote CRC chemoresistance.

We next further assessed whether hsa_circ_0004085 levels in the plasma of CRC patients were associated with oxaliplatin/5-FU response. Kaplan–Meier (KM) analysis indicated that high levels of hsa_circ_0004085 in plasma before therapy were associated with shorter progression-free survival (PFS) of CRC patients treated with therapeutic regimens based on XELOX or FOLFOX (Fig. 2F). There were no significant differences in clinical characteristics between patients in the hsa_circ_0004085 high and low groups before therapy (Table 1). These data suggested that high levels of hsa_circ_0004085 in the plasma of CRC patients were associated with a poor response to oxaliplatin/5-FU.

**Table 1:** Correlations of plasma hsa_circ_0004085 levels and clinical characteristics of 40 CRC patients

Variables	High plasma hsa_circ_0004085 (n = 20)	Low plasma hsa_circ_0004085 (n = 20)	p value
Gender			
Male	16	12	0.16752
Female	4	8	
Age			
≤ 60	12	7	0.11341
> 60	8	13	
Tumor size			
≤ 4 cm	8	10	0.52503
> 4 cm	12	10	
TNM stage			
I/II	0	0	N/A
III/VI	20	20	
Distant metastasis			
No	0	0	N/A
Yes	20	20	

### Fn infection increases exosome secretion of CRC cells and promotes enrichment of hsa_circ_0004085 in exosomes

**Fig. 3:**
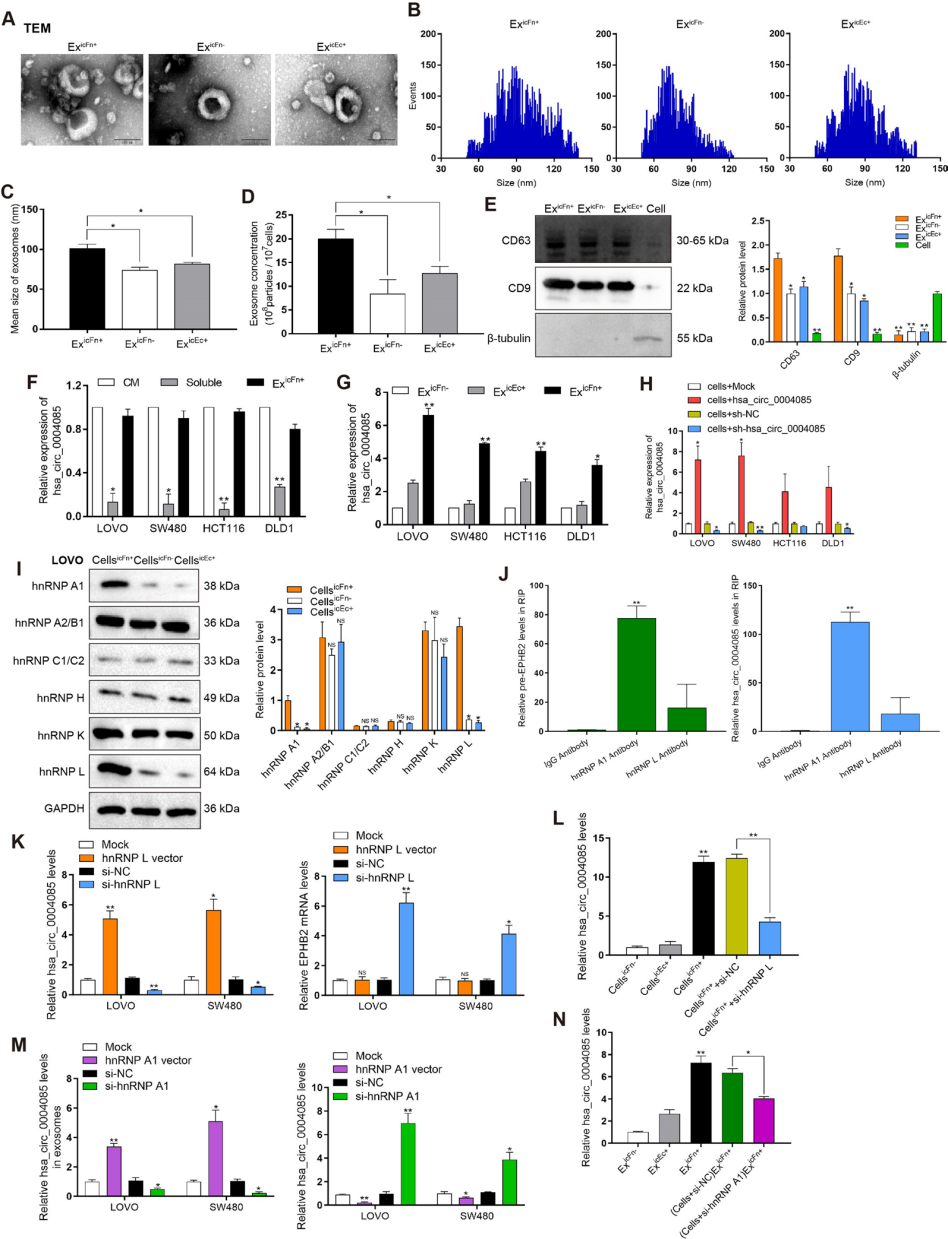
Fn infection increased exosomes secretion and promoted the enrichment of hsa_circ_0004085 in exosomes. **A** Morphologies of Ex^icFn+^, Ex^icFn−^, and Ex^icEc+^ were observed using TEM. **B**–**D** The size and number of Ex^icFn+^, Ex^icFn−^, and Ex^icEc+^ cells were analyzed by NTA. **E** Western blot detected protein levels of exosome-positive biomarkers (CD9 and CD63) and negative markers (β-tubulin) in Ex^icFn+^, Ex^icFn−^, Ex^icEc+^ and CRC cells. **F** The expression levels of hsa_circ_0004085 in culture medium, exosomes and culture medium without exosomes were determined by qRT-PCR. **G** The expression levels of hsa_circ_0004085 in Ex^icFn+^, Ex^icFn−^, and Ex^icEc+^ were determined by qRT-PCR. **H** The levels of hsa_circ_0004085 in CRC cells treated with lentiviral vector were determined by qRT-PCR. **I** Western blot analysis of the levels of core members in hnRNPs family in different group of LOVO cells. **J** RIP experiments tested the binding of hnRNPs to pre-EPHB2 or hsa_circ_0004085. **K** Changes in hsa_circ_0004085 or EPHB2 mRNA levels were determined by qRT-PCR after knockdown or overexpression of hnRNP L. **L** The expression levels of hsa_circ_0004085 in different group of LOVO cells were determined by qRT-PCR. **M** The expression levels of hsa_circ_0004085 in exosomes or cells in different group were determined by qRT-PCR. **N** The expression levels of hsa_circ_0004085 in exosomes from different groups were determined by qRT-PCR. (*P < 0.05, **P < 0.01, ***P < 0.001, NS: not signifcant)

CRC cells were infected with Fn at an MOI of 10:1 (bacteria: cells), and CRC cells intracellularly infected with Fn (Cells^icFn+^) were obtained after the exchange of cell medium (CM). Exosomes of living Fn-infected cells (Ex^icFn+^) were isolated from the medium after 48 h of continued culture, and exosomes derived from heat-inactivated Fn-infected cells (Ex^icFn−^) and exosomes derived from *Escherichia coli*-infected cells (Ex^icEc+^) were used as controls. Transmission electron microscopy (TEM) revealed that these purified vesicle samples were oval and globular (Fig. 3A). Nanoparticle tracking analysis (NTA) results showed that Ex^icFn+^ was larger in size and higher in concentration than Ex^icFn−^ or Ex^icEc+^ (Fig. 3B–D). In addition, the presence of positive biomarkers for exosomes (CD9 and CD63) was verified in Ex^icFn+^, Ex^icFn−^, and Ex^icEc+^ collected from the same volume of culture supernatant by Western blotting, whereas negative markers (β-Tubulin) were not detected. Further analysis showed that Fn infection significantly increased exosome secretion by CRC cells (Fig. 3E). Interestingly, the hsa_circ_0004085 level in exosomes was almost equal to that in whole CM of CRC^icFn+^, indicating that exosomes are the main carrier of extracellular hsa_circ_0004085 (Fig. 3F). Moreover, the level of hsa_circ_0004085 was significantly higher in Ex^icFn+^ than in Ex^icFn−^ or Ex^icEc+^ (Fig. 3G). We then constructed LOVO and SW480 cell lines with stable overexpression or knockdown of hsa_circ_0004085 for subsequent experiments (Fig. 3H). The above data suggest that Fn infection increases exosome secretion by CRC cells and promotes the packaging of hsa_circ_0004085 into exosomes.

The steady-state abundance of circRNAs is thus a balanced result of the efficiency of circRNA production, nuclear export and turnover. Among regulators that affect these procedures, heterogeneous nuclear ribonucleoproteins (hnRNPs) family members can not only influence circRNA abundance but also regulate exosome assembly [23]. To explore how Fn infection upregulated the levels of circRNA in intracellular and exosomes simultaneously, we examined the expression level of core members in hnRNPs family and found that hnRNP L and hnRNP A1 were significantly upregulated in CRC cells infected with Fn (Fig. 3I). It has been reported that hnRNP L promotes circRNAs formation through regulating reverse splicing of pre-mRNA, while hnRNP A1 is able to load the non-coding RNA into exosomes [24, 25]. RNA immunoprecipitation (RIP) was performed using hnRNP L or hnRNP A1 antibodies, and qRT-PCR revealed hnRNP L binding to pre-EPHB2 and hnRNP A1 binding to hsa_circ_0004085 (Fig. 3J). Next, we overexpressed hnRNP L in CRC cells and found that hsa_circ_0004085 increased, but EPHB2 was uninfluenced. We silenced hnRNP L and found that hsa_circ_0004085 decreased, but EPHB2 increased (Fig. 3K). Increase of hsa_circ_0004085 induced by Fn infection in CRC cells was partially reversed by knockdown of hnRNP L (Fig. 3L). Additionally, overexpression of hnRNP A1 increased exosomal hsa_circ_0004085 enrichment but decreased its level in CRC cells. Knockdown of hnRNP A1 increased hsa_circ_0004085 in CRC cells but decreased its level in exosome (Fig. 3M). Enrichment of hsa_circ_0004085 in exosome induced by Fn infection was partially reversed by knockdown of hnRNP A1 (Fig. 3N). These results suggested that hnRNP L induced by Fn infection promoted hsa_circ_0004085 formation through pre-EPHB2 reverse splicing, and that hsa_circ_0004085 was packaged into exosomes by hnRNP A1.

### Intercellular transfer of hsa_circ_0004085 mediated by Ex^icFn+^ propagates oxaliplatin or 5-FU resistance by relieving ER stress in vitro

To verify whether hsa_circ_0004085 delivered by Ex^icFn+^ is associated with the chemotherapy response at the cellular level, we first treated different groups of CRC cells with different concentrations of oxaliplatin or 5-FU. The results showed that, compared with the control (Cells^icFn−^, Cells^icEc+^), the viability of cells^icFn+^ treated with oxaliplatin/5-FU increased significantly, and the 50% inhibitory concentration (IC50) of oxaliplatin/5-FU for cells^icFn+^ increased (Additional file 1: Fig. S2A). LOVO and SW480 cells infected with living Fn were treated with oxaliplatin or 5-FU and used to perform CCK-8 assays, colony formation assays, and EDU experiments. Fn infection significantly reduced the cytotoxicity induced by oxaliplatin/5-FU, whereas knockdown of hsa_circ_0004085 abolished this effect (Additional file 1: Fig. S2B-S2D). As shown in Additional file 1: Fig. S3A, overexpression of hsa_circ_0004085 conferred tolerance to oxaliplatin/5-FU in CRC cells, while knockdown of hsa_circ_0004085 increased cell sensitivity to drugs. The above data confirmed that hsa_circ_0004085 was sufficient to confer resistance to oxaliplatin or 5-FU in CRC cells.

**Fig. 4:**
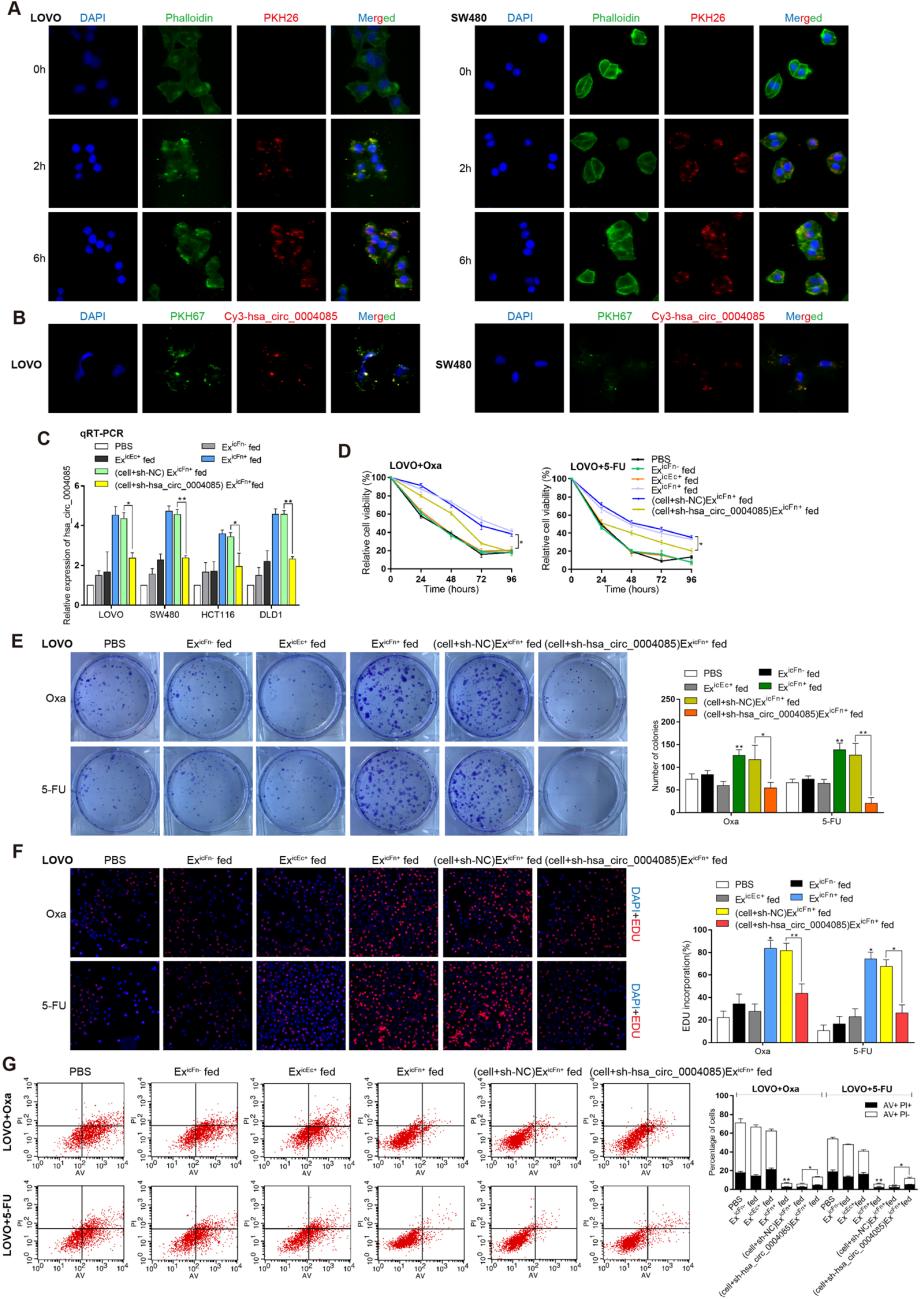
Ex^icFn+^ transmitted resistance to oxaliplatin/5-FU by delivering hsa_circ_0004085 in vitro. **A** Tracer analysis of exosomes recorded the uptake of 10 µg PKH26 (red)-stained Ex^icFn+^ by 1 × 10^5^ cells after incubation together for 0 h, 2 h and 6 h. DAPI (blue): cell nucleus, Phalloidin (green): cytoskeleton. **B** CRC cells were transfected with the Cy3-tagged hsa_circ_0004085 expression vector (orange-red), and exosomes in the medium were isolated and labeled with PKH67 (green). The location of Cy3-hsa_circ_0004085 and PKH67-labeled exosomes was observed after coincubation with recipient CRC cells. **C** The expression levels of hsa_circ_0004085 in CRC cells incubated with Ex^icFn+^, Ex^icFn−^ or Ex^icEc+^ were determined by qRT-PCR. **D**–**F** Resistance to Oxa/5-Fu of LOVO cells incubated directly with Ex^icFn+^, Ex^icFn−^ or Ex^icEc+^ was analyzed by CCK-8 analysis, colony formation and EdU experiments. **G** Oxa/5-Fu-induced apoptosis of LOVO cells incubated directly with Ex^icFn+^, Ex^icFn−^ or Ex^icEc+^ was tested with flow cytometry. (*P < 0.05, **P < 0.01, ***P < 0.001, NS: not signifcant)

Subsequently, to determine whether Ex^icFn+^ could be taken up by CRC cells, 1 × 10^5^ LOVO cells and 10 μg PKH26 (red)-stained Ex^icFn+^ were incubated together for 0 h, 2 h and 6 h. Confocal imaging showed that Ex^icFn+^ was indeed taken up by LOVO cells after 6 h of incubation, and similar results were also obtained in SW480 cells (Fig. 4A). To further confirm whether hsa_circ_0004085 could be transferred to recipient cells via exosomes, we labeled the hsa_circ_0004085 vector with Cy3 (orange red) and transfected CRC cells with the vector. Exosomes were then isolated from CM and labeled with PKH67 (green). As shown in Fig. 4B, after incubation with the labeled exosomes, colocalization of Cy3-hsa_circ_0004085 and PKH67 was observed in most recipient cells, indicating that hsa_circ_0004085 was transferred from cells^icFn+^ to uninfected CRC cells via exosomes. CRC cells were subsequently incubated with CM of CRC^icFn+^ cells (exosomes were physically removed or not removed). The qRT-PCR results showed that CRC cells grown in CM from CRC^icFn+^ cells (CM^icFn+^) expressed higher levels of hsa_circ_0004085. However, there was no significant change in the expression of hsa_circ_0004085 in CRC cells cultured in CM^icFn+^ after exosome removal (Additional file 1: Fig. S3B). Similarly, higher levels of hsa_circ_0004085 were detected in CRC cells cocultured with CRC^icFn+^ cells but not CRC^icFn+^ cells that had been pretreated with GW4869 for pharmacological depletion of exosomes (Additional file 1: Fig. S3C). The level of intracellular hsa_circ_0004085 increased after incubation with Ex^icFn+^ and could be partially counteracted by knockdown of hsa_circ_0004085 in recipient cells (Fig. 4C). These findings revealed that hsa_circ_0004085 can be transferred from CRC^icFn+^ cells into CRC recipient cells via exosomes.

To investigate the effect of intercellular transfer of hsa_circ_0004085 on CRC chemoresistance in vitro, we performed CCK8, colony formation and EdU experiments. As shown in Fig. 4D–F, LOVO cells incubated with Ex^icFn+^ showed reduced sensitivity to oxaliplatin/5-FU. Similar results were obtained in SW480 cells (Additional file 1: Fig. S3D-S3F). Flow cytometry analysis revealed that incubation with Ex^icFn+^ inhibited apoptosis induced by oxaliplatin/5-FU in recipient cells (Fig. 4G and Additional file 1: Fig. S3G). These effects could be partially abrogated by sh-hsa_circ_0004085 in recipient cells (Fig. 4D–G and Additional file 1: Fig. S3D-S3G). The above results indicated the critical role of exosomal hsa_circ_0004085 in drug-resistant transmission.

**Fig. 5:**
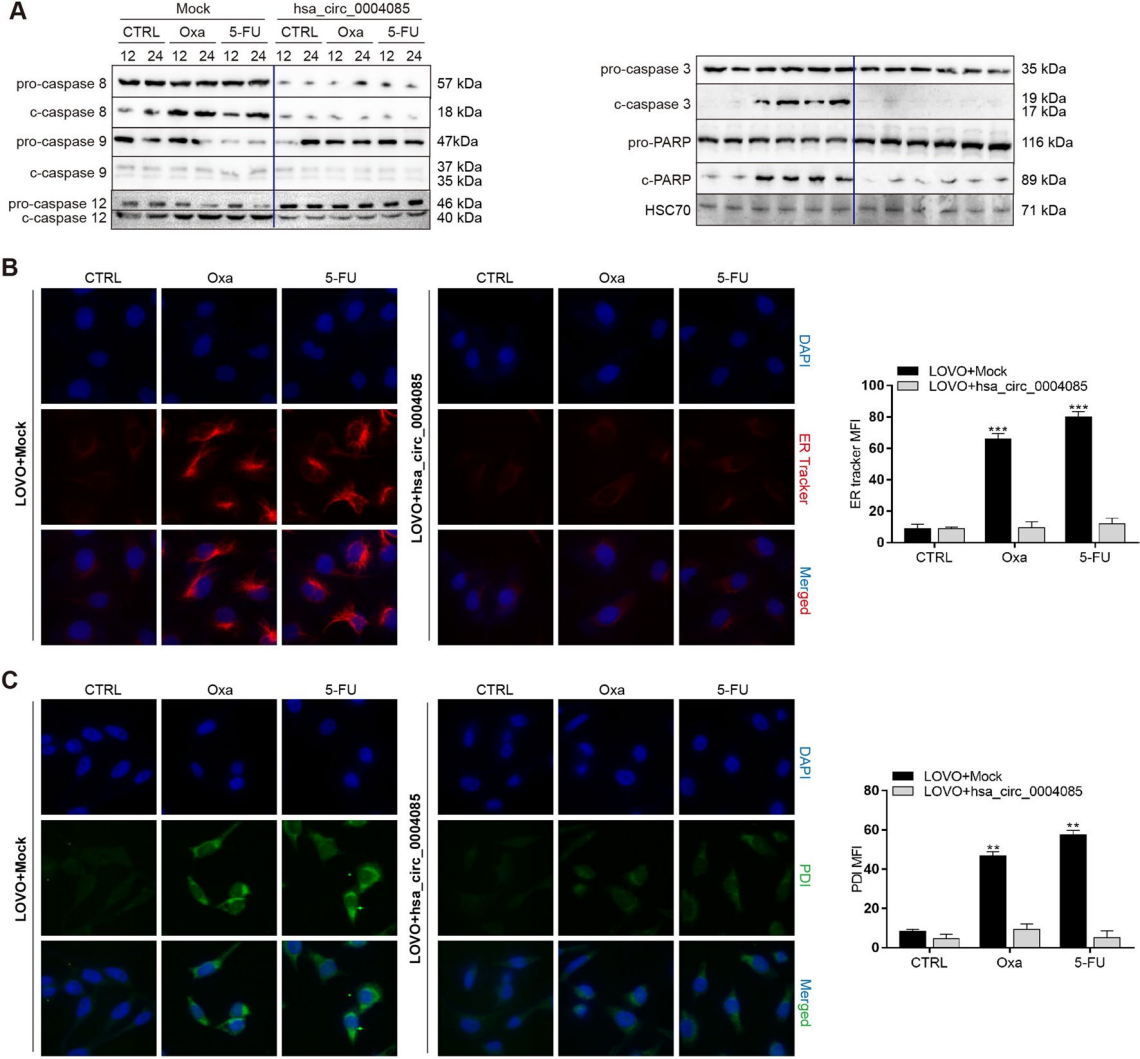
Hsa_circ_0004085 relieved 5-Fu/Oxa-induced ER stress, thereby conferring drug resistance to CRC cells. **A** Western blot analysis of the influence of hsa_circ_0004085 on key markers of the cell death pathway induced by Oxa/5-Fu. **B** ER tracking analysis examined the influence of hsa_circ_0004085 on ER-tracker MFI in LOVO cells treated with Oxa/5-Fu. **C** IF was used to examine the influence of hsa_circ_0004085 on the PDI MFI in LOVO cells treated with Oxa/5-Fu. (*P < 0.05, **P < 0.01, ***P < 0.001, NS: not signifcant)

We next investigated the effect of hsa_circ_0004085 on key molecules of cell death pathways induced by oxaliplatin/5-FU. As shown in Fig. 5A, overexpression of hsa_circ_0004085 in LOVO cells significantly reduced the levels of c-caspase 8, c-caspase-12, c-caspase 3 and poly ADP ribose polymerase (PARP). We hypothesized that hsa_circ_0004085 overexpression might alleviate oxaliplatin/5-FU-induced ER stress because caspase-12 activation is a key marker of ER stress pathways. The feature of cells experiencing ER stress is an enlarged endoplasmic reticulum lumen. As shown in Fig. 5B, overexpression of hsa_circ_0004085 was associated with a decrease in the median fluorescence intensity (MFI) of the ER tracker after chemotherapy, indicating that ER stress was alleviated. In addition, we performed immunofluorescence (IF) experiments aimed at protein disulfide isomerase (PDI), an ER stress marker protein, and found that PDI expression increased only in cells with low hsa_circ_0004085 expression (Fig. 5C), reinforcing the notion that hsa_circ_0004085 overexpression can reduce chemotherapy-induced ER stress.

To further verify whether hsa_circ_0004085 regulates chemoresistance through the ER stress pathway, we treated CRC cell lines overexpressing hsa_circ_0004085 with oxaliplatin/5-FU in the presence of the ER stress inducer tunicamycin (TM). As shown in Additional file 1: Fig. S4A and S4B, TM almost completely abolished oxaliplatin/5-FU resistance induced by hsa_circ_0004085.

### Intercellular transfer of hsa_circ_0004085 mediated by Ex^icFn+^ propagates oxaliplatin or 5-FU resistance by relieving ER stress in vivo

**Fig. 6:**
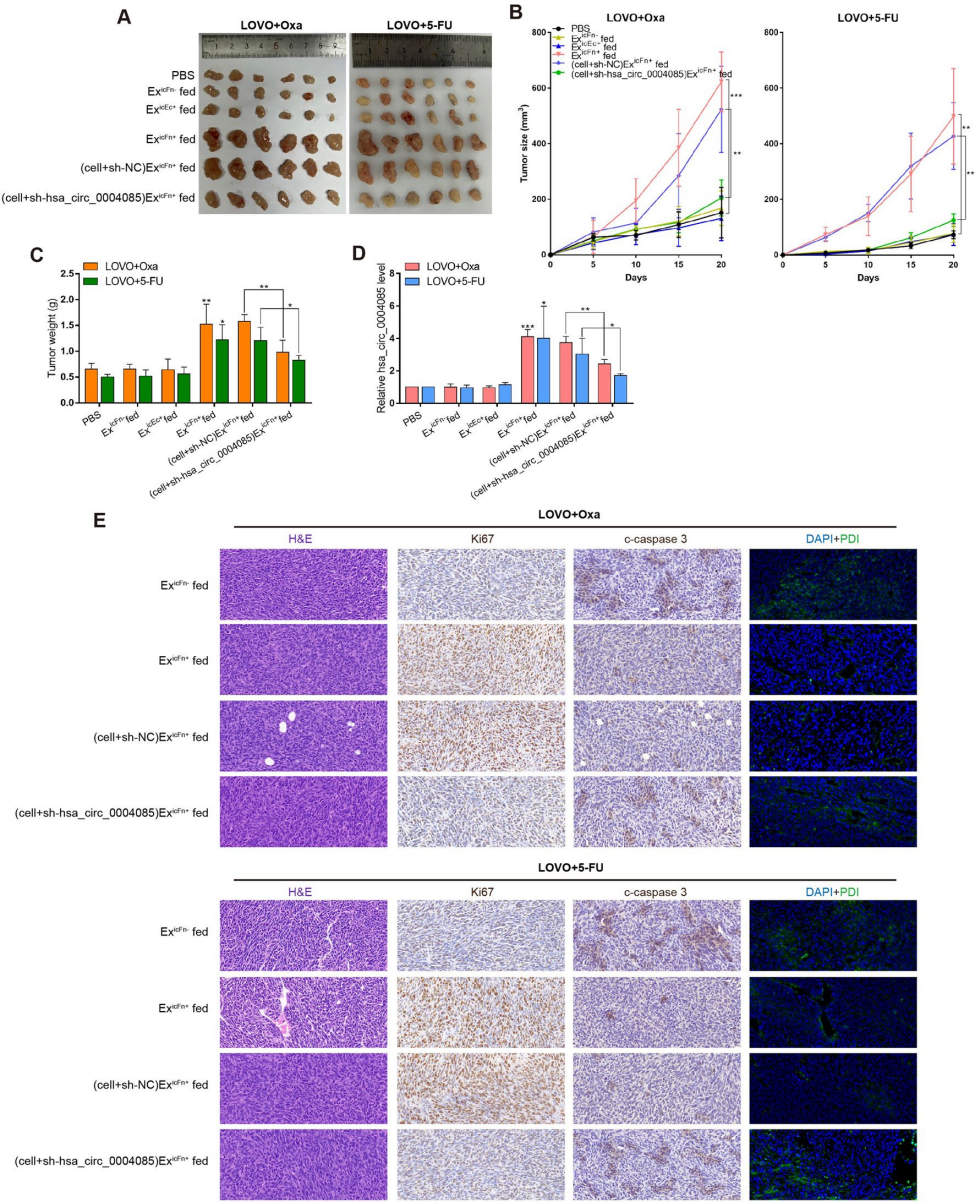
Ex^icFn+^ transmitted resistance to oxaliplatin/5-FU by delivering hsa_circ_0004085 in vivo. **A**, **B** Ex^icFn+^, Ex^icFn−^ or Ex^icEc+^ were injected into CRC xenografts, and the mice were intraperitoneally injected with Oxa or 5-FU. The growth of subcutaneous tumors was measured and recorded, and the mice were finally sacrificed to harvest the subcutaneous tumors. **C** Subcutaneous tumors from different groups were weighed to assess their responsiveness to Oxa/5-FU. **D** Total RNA was extracted from subcutaneous tumor tissues of different groups, and the expression levels of hsa_circ_0004085 were determined by qRT-PCR. **E** H&E staining of subcutaneous tumors was carried out using hematoxylin (bluish violet) and eosin (red). IHC of subcutaneous tumors was carried out using ki-67 and c-caspase 3 antibodies, while IF was performed by using anti-PDI antibody (green). (*P < 0.05, **P < 0.01, ***P < 0.001, NS: not signifcant)

To demonstrate the effect of exosomal hsa_circ_0004085 on the CRC response to oxaliplatin or 5-FU in vivo, we intratumorally injected exosomes into xenografts and confirmed that exosomes carrying hsa_circ_0004085 can enter CRC cells in subcutaneous tumor (Additional file 1: Fig. S4C). As shown in Fig. 6A–D, Ex^icFn+^, but not Ex^icFn−^ or Ex^icEc+^, significantly impaired the sensitivity of CRC xenografts to oxaliplatin/5-FU and increased the levels of hsa_circ_0004085 in the tumors, indicating that Ex^icFn+^ could transmit hsa_circ_0004085 and oxaliplatin/5-FU resistance in vivo. The results of immunohistochemistry (IHC) suggested that Ex^icFn+^ conferred tolerance of CRC xenografts to oxaliplatin/5-FU and inhibited apoptosis induced by oxaliplatin/5-FU. Immunofluorescence (IF) analysis showed that Ex^icFn+^ alleviated ER stress induced by oxaliplatin/5-FU in vivo (Fig. 6E). However, these effects of Ex^icFn+^ could all be abolished by sh-hsa_circ_0004085 in recipient cells (Fig. 6A–E). Collectively, these findings suggest that Ex^icFn+^ can propagate drug resistance by relieving ER stress induced by oxaliplatin or 5-FU in recipient CRC cells in vivo through intercellular transfer of hsa_circ_0004085.

### Hsa_circ_0004085 alleviates ER stress by regulating key molecules in the UPR

**Fig. 7:**
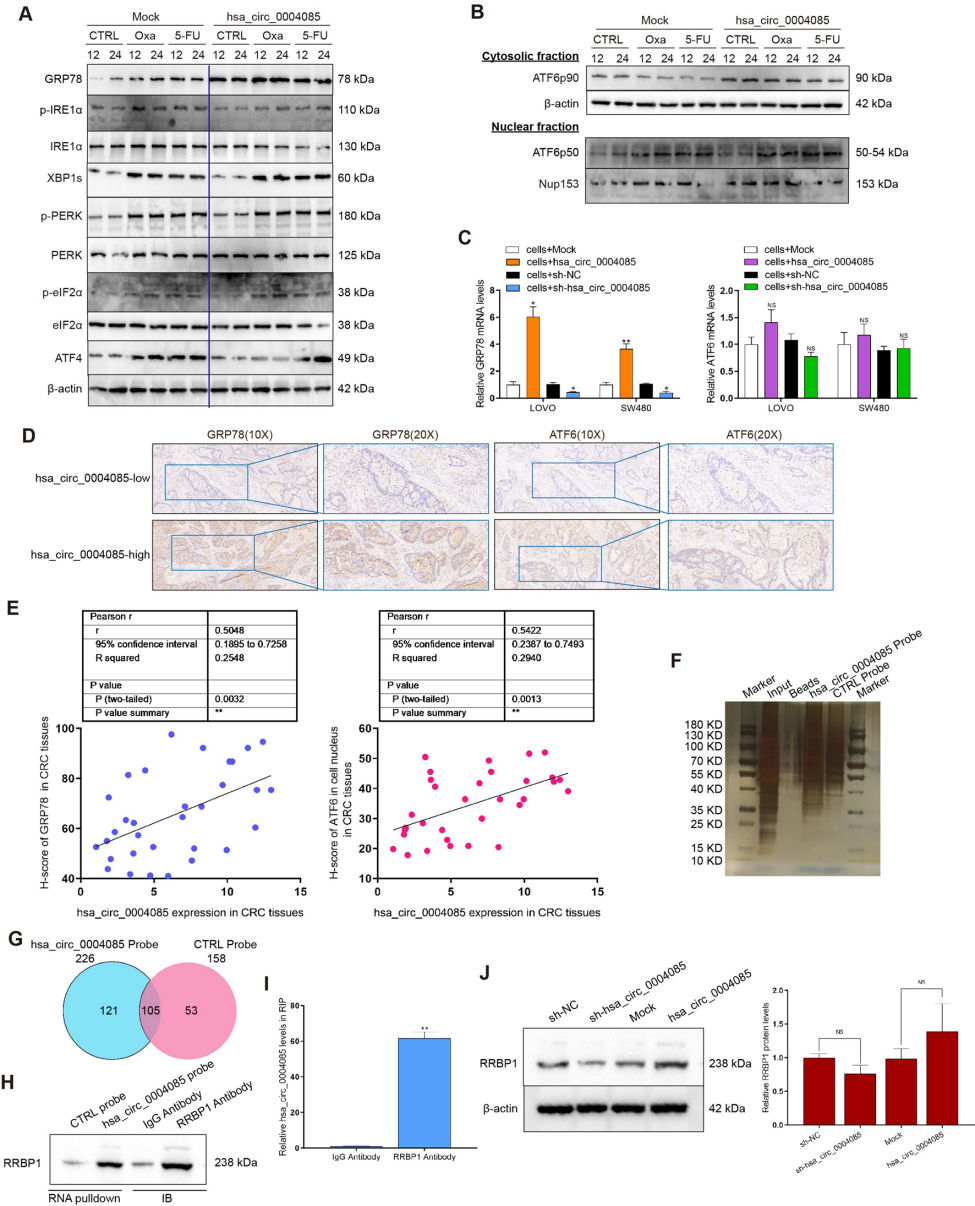
Hsa_circ_0004085 regulated key molecules in the UPR. **A**, **B** Western blot analysis of the levels of UPR-associated proteins in LOVO cells treated with Oxa and 5-FU. **C** Changes in GRP78 mRNA and ATF6 mRNA levels were determined by qRT-PCR after knockdown or overexpression of hsa_circ_0004085. **D** The protein level and distribution of GRP78 and ATF6 in CRC tissues with high or low hsa_circ_0004085 expression levels were detected by IHC (serial sections). **E** The correlation between hsa_circ_0004085 expression and the H-score of GRP78 and nuclear ATF6 (ATF6p50) was analyzed in CRC tumor tissues. **F** The pull-down products of hsa_circ_0004085 and the control probe were subjected to silver staining. **G** LC–MS/MS analysis of the pull-down products of hsa_circ_0004085 and the control probe. **H** The products of RNA pulldown and IB were analyzed by Western blot with anti-RRBP1 antibody. **I** Hsa_circ_0004085 was measured by qRT-PCR in immunoprecipitates of RRBP1. **J** Western blotting was used to detect the influence of hsa_circ_0004085 on RRBP1. (*P < 0.05, **P < 0.01, ***P < 0.001, NS: not signifcant)

Normally, the molecular chaperone binding immunoglobulin protein (BIP, also known as GRP78 or HSPA5) clasps on transmembrane proteins in the ER membrane to maintain homeostasis. When factors inside and outside the cell disrupt the protein folding capacity of the ER, leading to ER stress, the unfolded protein response (UPR) is initiated in an attempt to restore ER homeostasis and promote tumor cell adaptation to various injuries [26]. Considering the dependency of the effects induced by hsa_circ_0004085 on ER stress alleviation, we focused on the expression of UPR-related proteins in LOVO cells treated with oxaliplatin/5-FU. As shown in Fig. 7A and B, Western blot analysis showed that GRP78 and nuclear ATF6 (ATF6p50) were upregulated in cells overexpressing hsa_circ_0004085, but the phosphorylation levels of IRE1 and PERK were not affected by hsa_circ_0004085, as were the expression levels of XBP1s and ATF4. As shown in Fig. 7C, qRT-PCR confirmed that overexpression of hsa_circ_0004085 upregulated GRP78 mRNA levels, while knockdown of hsa_circ_0004085 downregulated GRP78 mRNA levels. However, hsa_circ_0004085 showed no effect on the level of ATF6 mRNA in CRC cells. Hsa_circ_0004085 was positively correlated with GRP78 mRNA levels but not ATF6 mRNA levels in CRC tissues (Additional file 1: Fig. S4D). Sequencing data from The Cancer Genome Atlas (TCGA) and Genotype-Tissue Expression (GTEX) were analyzed using the Gene Expression Profiling Interactive Analysis (GEPIA2) platform, and the results showed that GRP78 levels were increased in multiple tumors, including colon cancer (Additional file 1: Fig. S4E). IHC in CRC tissues revealed that the GRP78 protein level in the hsa_circ_0004085 high-expression group was much higher than that in the hsa_circ_0004085 low-expression group, and a positive correlation was observed between them (Fig. 7D, E). The nuclear ATF6p50 level was upregulated in tumor tissues of the hsa_circ_0004085 high-expression group, and the hsa_circ_0004085 level was positively correlated with the ATF6p50 level (Fig. 7D, E). These data suggest that hsa_circ_0004085 alleviates ER stress by driving some key molecules in the UPR process in CRC cells.

### Hsa_circ_0004085 enhances the stability of GRP78 mRNA by binding RRBP1

To further investigate the mechanism by which hsa_circ_0004085 delivered to recipient CRC cells exerts its effects, we performed liquid chromatography tandem mass spectrometry (LC–MS/MS) analysis of the pull-down samples of hsa_circ_0004085 and compared them to controls (Additional file 1: Fig. S5A and Fig. 7F). As a result, 121 binding proteins of hsa_circ_0004085 were identified (Fig. 7G) and are listed in Additional file 3: Table S2. Gene Ontology (GO) analysis showed that these binding proteins were associated with multiple functions, such as mRNA metabolic process and ribonucleoprotein complex binding (Additional file 1: Fig. S5B). Kyoto Encyclopedia of Genes and Genomes (KEGG) database analysis suggested that these binding proteins are mainly located in pathways such as the spliceosome and protein processing in the ER (Additional file 1: Fig. S5B). ProteinPilot was used to analyze the LC–MS/MS data, and ribosome-binding protein 1 (RRBP1) had the highest score. Among the ten highest-scoring pull-down products, we found the hnRNP A1 mentioned above, further confirming the binding of hsa_circ_0004085 to hnRNP A1. RRBP1, a ribosomal receptor, mediates the interaction between the ribosome and the ER membrane (UniProt, https://www.uniprot.org/uniprot/Q9P2E9#entryinformation). It has been reported to alleviate ER stress, thus alleviating apoptosis and stimulating cell proliferation [27, 28]. To further validate the interaction between hsa_circ_0004085 and RRBP1, we performed Western blotting and qRT-PCR with pulldown products of hsa_circ_0004085. The results showed that biotin-labeled hsa_circ_0004085 probes pulled down RRBP1 and hsa_circ_0004085 from LOVO cell extracts (Fig. 7H and Additional file 1: Fig. S5C). Hsa_circ_0004085 and RRBP1 were also detected in RRBP1 immunoprecipitates by performing RIP and immunoblotting (IB) of the RNA-RRBP1 complex using anti-RRBP1 antibody (Additional file 1: Fig. S5D, Fig. 7H and I). Furthermore, western blot and qRT-PCR verified that knockdown or overexpression of hsa_circ_0004085 had no obvious effect on the expression of RRBP1 (Fig. 7J and Additional file 1: Fig. S5E). qRT-PCR showed that overexpression or knockdown of RRBP1 had no obvious effect on the level of hsa_circ_0004085 (Additional file 1: Fig. S5F and S5G). Our results demonstrate that hsa_circ_0004085 binds to RRBP1, but they do not affect each other in terms of expression levels.

**Fig. 8:**
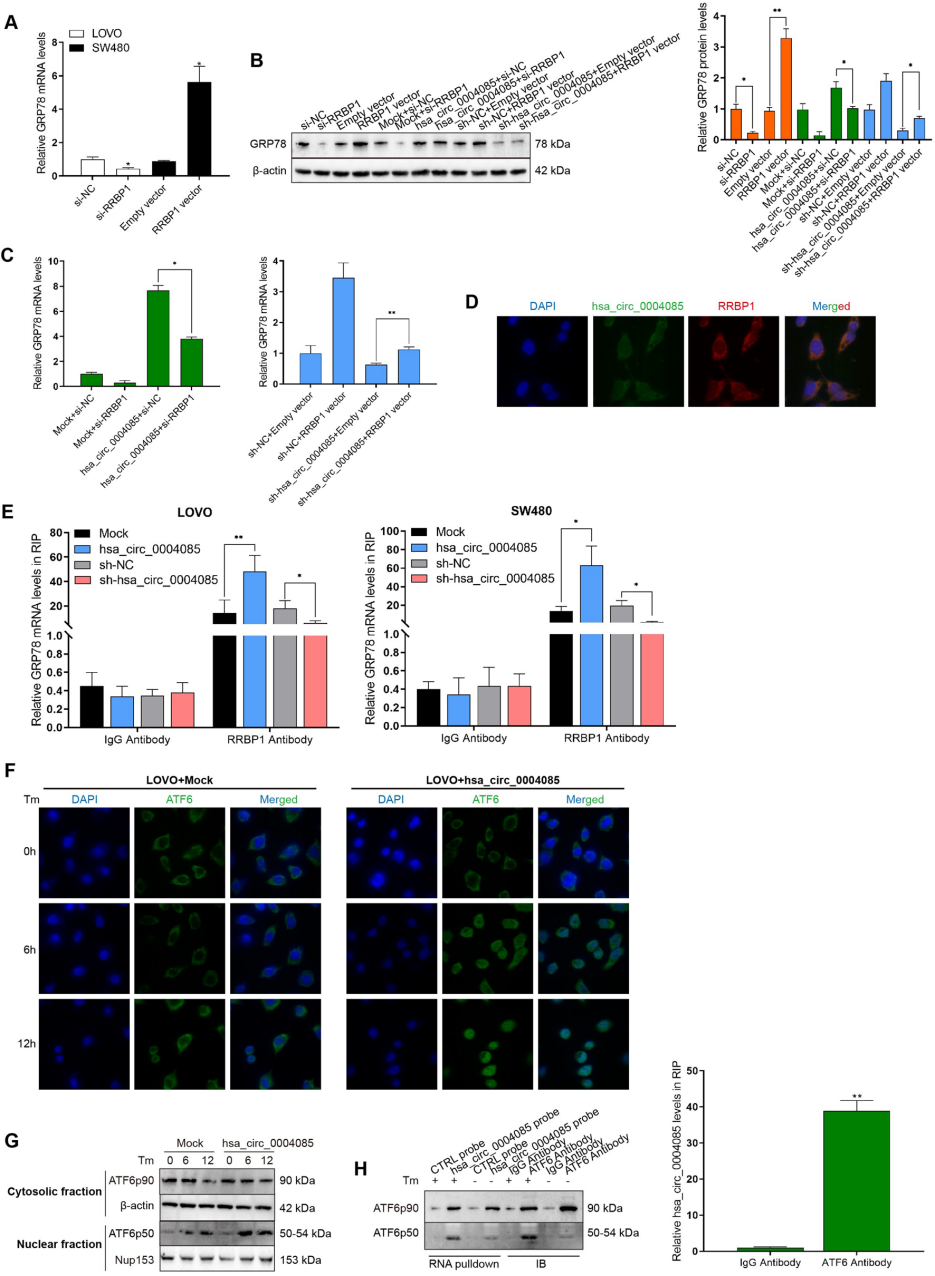
Regulatory mechanism of hsa_circ_0004085 to GRP78 and ATF6. **A** QRT-PCR detected the influence of RRBP1 on GRP78 mRNA. **B** Western blot analysis of the influence of hsa_circ_0004085 and RRBP1 on GRP78. **C** QRT-PCR detected the influence of the hsa_circ_0004085 vector and si-RRBP1 on GRP78 mRNA (left). qRT-PCR was used to detect the influence of sh-hsa_circ_0004085 and RRBP1 vectors on GRP78 mRNA (right). **D** Subcellular localization of hsa_circ_0004085 and RRBP1 was detected by FISH combined with IF. **E** RIP experiments tested the effect of hsa_circ_0004085 on the binding of RRBP1 to GRP78 mRNA. **F** IF showed the effect of hsa_circ_0004085 on the nuclear translocation of ATF6p50 in LOVO cells. **G** Western blot analysis of the effect of hsa_circ_0004085 on the nuclear expression of ATF6p50 in LOVO cells. **H** The pulldown products of the hsa_circ_0004085 probe and IB products of the anti-ATF6 antibody were analyzed by Western blotting (left). Hsa_circ_0004085 was measured by qRT-PCR in immunoprecipitates of ATF6. (*P < 0.05, **P < 0.01, ***P < 0.001, NS: not signifcant)

Analysis of data from TCGA and GTEX revealed increased levels of RRBP1 in multiple tumors, including colon cancer (Additional file 1: Fig. S6A). The overall survival of patients with higher RRBP1 levels was poorer than that of patients with lower RRBP1 levels (Additional file 1: Fig. S6B). In addition, the expression level of RRBP1 was positively correlated with that of GRP78 (Additional file 1: Fig. S6C). RRBP1 reportedly alleviates ER stress-induced apoptosis in lung cancer cells by enhancing GRP78 expression [27]. To investigate whether hsa_circ_0004085 regulates the level of GRP78 in CRC cells by binding RRBP1, we detected the expression of GRP78 at the mRNA and protein levels and observed that si-RRBP1 decreased the expression of GRP78, whereas overexpression of RRBP1 resulted in the upregulation of GRP78 (Fig. 8A and B). Overexpression of RRBP1 partially reversed GRP78 downregulation induced by sh-hsa_circ_0004085, while knockdown of RRBP1 partially reversed GRP78 upregulation induced by hsa_circ_0004085 (Fig. 8B and C). As shown in Fig. 8D, FISH and IF experiments revealed that the colocalization of hsa_circ_0004085 and RRBP1 mainly occurred in the cytoplasm, suggesting that their regulatory effect on GRP78 mainly occurs at the posttranscriptional level.

It has been reported that RRBP1 promotes mRNA stability by recruiting mRNAs to anchor in the ER [29, 30]. We therefore tested the stability of GRP78 mRNA treated with actinomycin D, and the results showed that the half-life of GRP78 mRNA was correlated with the expression of RRBP1 (Additional file 1: Fig. S7A). Therefore, we hypothesized that hsa_circ_0004085 may promote GRP78 mRNA stability by recruiting RRBP1. To test this hypothesis, we performed RIP experiments subsequent to knockdown or overexpression of hsa_circ_0004085 and observed that binding of RRBP1 to GRP78 mRNA was affected by hsa_circ_0004085 (Fig. 8E). Then, LOVO and SW480 cells with knockdown of hsa_circ_0004085 were cotransfected with the RRBP1 vector and showed that RRBP1 partially abolished the shortening effect of sh-hsa_circ_0004085 on the half-life of GRP78 mRNA (Additional file 1: Fig. S7B). In contrast, si-RRBP1 partially attenuated the prolonged effect of hsa_circ_0004085 on the half-life of GRP78 mRNA (Additional file 1: Fig. S7C). Taken together, hsa_circ_0004085 increases the stability of GRP78 mRNA by binding to RRBP1.

### Hsa_circ_0004085 promotes nuclear translocation of ATF6p50

To further explore the way in which hsa_circ_0004085 upregulated ATF6p50 in the nucleus, IF and Western blotting were carried out to assess the subcellular localization of ATF6 in LOVO cells induced with TM for the indicated times. The IF results showed that the nuclear translocation of ATF6p50 was significantly accelerated in LOVO cells overexpressing hsa_circ_0004085 (Fig. 8F). Western blot analysis showed that nuclear translocation of ATF6p50 was significantly enhanced by hsa_circ_0004085 (Fig. 8G). As shown in Fig. 8H, the hsa_circ_0004085 probe was able to pull down ATF6p50 in LOVO cells stimulated with TM, while ATF6p90, ATF6p50 and hsa_circ_0004085 were immunoprecipitated by the anti-ATF6 antibody. Taken together, hsa_circ_0004085 promoted the nuclear translocation of ATF6p50 in CRC cells.

## Discussion

Colon cancer is the third most common cancer in the world, with an incidence of 10.0% and a mortality rate of 9.4% of all cancers [31]. Fluorouracil, oxaliplatin, and irinotecan form the chemotherapy backbone in different iterations of chemotherapy regimens for colon cancer [32, 33]. Immune checkpoint inhibitors (ICIs) are one of the new options for CRC patients, a strategy that exploits the patient's own immune system to combat cancer cells. Despite the success of ICIs in recent years, their benefit in metastatic CRC is limited to 3–7% of cases with microsatellite instability [3]. Additionally, the side effects of this therapy can be very risky for patients if they occur [34]. Fluorouracil and oxaliplatin remain the most commonly used baseline chemotherapeutic agents for patients with CRC. The initial response of CRC patients to chemotherapy is usually effective, but many patients eventually experience tumor progression due to drug resistance [6]. Therefore, understanding the mechanisms of chemoresistance in CRC is essential for optimizing current therapeutic strategies. It has been reported that cancer therapies accentuate tumor cell stresses while tumor ecosystems, including cancer cells, immune cells, and stroma adapt to therapeutic stresses in three different ways: (a) interdict stress mitigation to induce cell death, (b) increase stress to induce cellular catastrophe and (c) exploit emergent vulnerabilities in tumor and microenvironment cells [35]. Among them, aberrant activation of ER stress sensors and their downstream signalling pathways have emerged as key regulators of tumour growth and metastasis as well as response to chemotherapy [26]. There are three ER stress sensors: IRE-1 (inositol-requiring protein 1), PERK (PKR-like ER kinase) and ATF6 (activating transcription factor). During ER stress, GRP78 dissociates from the sensors and activates the latter to initiate the UPR [27].

Sequencing studies in recent years have demonstrated microbial composition and ecological changes in CRC patients, while the role of several bacteria (*Fusobacterium nucleatum* included) in colorectal carcinogenesis has been identified [36]. Moreover, the gut microbiota influences the host response to chemotherapy [37]. However, we know little about the potential role of the gut microbiota in CRC chemoresistance based on recent findings that the tumor microenvironment and fecal samples of CRC patients are enriched with Fn, which has been suggested as a risk factor for CRC development and progression [38]. Exosomes from Fn-infected CRC cells promote tumor metastasis by selectively carrying miR-1246/92b-3p/27a-3p and CXCL16, Fn promotes CRC metastasis by regulating KRT7-AS/KRT7, and Fn promotes CRC development by activating the cytochrome P450 and epoxyoctadecenoic acid axes [11, 39, 40]. Humans have a whole new understanding of the role of Fn in the development and progression of CRC, but it remains to be fully explored how Fn mediates chemoresistance in CRC. In this study, we detected abnormally elevated levels of hsa_circ_0004085 in tumor tissues and plasma of colon cancer patients with positive intracellular Fn infection. Moreover, high levels of hsa_circ_0004085 expression in plasma were predictive of poor clinical response to fluorouracil or oxaliplatin. Our data may indirectly explain why Fn infection is associated with worse clinical outcomes in CRC. Since the number of Fn is associated with the risk of CRC recurrence, the detection of Fn after surgery may be an effective way to predict the outcome of chemotherapy in patients. The results of the present study may provide insights for future research and clinical work to develop strategies for the prevention and combined treatment of CRC by targeting the microbiota.

Most cell types release extracellular vesicles (EVs), and EVs with a diameter of 30–150 nm released through the endocytic pathway are defined as exosomes. Bacterial infection affects the release of exosomes, and the cargo loaded in exosomes may exert regulatory effects on recipient cells [41]. For example, exosomes released from macrophages infected by *mycobacteria* contain mycobacterial antigens to stimulate immune responses [42]. Exosomes released from *Salmonella*-infected macrophages have proinflammatory effects [43]. During infection with uropathogenic *Escherichia coli*, exosomes released by urothelial cells are able to recruit mast cells and induce bladder urothelial barrier dysfunction [44]. These reports suggest that bacterial infection induces the secretion of exosomes from host cells to alter immune responses and pathophysiological processes. Exosomes carry not only proteins, DNA, microRNAs, and lncRNAs, but also circRNAs. As a new type of non-coding RNA (ncRNAs), circRNA is structurally stable and hard to be degraded [17]. CircRNAs carried by exosomes play a crucial part in CRC: exosomal circPACRGL promoted CRC cell proliferation, migration and invasion, as well as differentiation of N1 to N2 neutrophils, CRC cell excreted out tumor-suppressive circRHOBTB3 via exosome to sustain its fitness, exosomal circTUBGCP4 promotes vascular endothelial cell tipping and CRC metastasis by activating Akt signaling pathway [45–47]. The present study found that Fn infection promoted hsa_circ_0004085 formation by hnRNP L and packaged hsa_circ_0004085 into exosomes by hnRNP A1. Such exosomes loaded with hsa_circ_0004085 were transferred between cells and then entered recipient cells, and hsa_circ_0004085 was released to alleviate ER stress and thus spread oxaliplatin or 5-FU resistance by regulating the expression level of GRP78 and the nuclear translocation of ATF6p50. Therefore, circulating exosomes have a potential diagnostic effect on the microbial infection status of intestinal tissue and may have a predictive role in chemoresistance in colon cancer patients. Whether intercepting exosomes or targeting exosomal contents in the circulation plays a role in inhibiting the delivery of drug resistance, preventing distant metastasis of tumors, or avoiding immune escape of tumor cells is a worthwhile direction.

CircRNAs have long been considered ncRNAs with regulatory potency, such as bind to the host genes at their synthesis locus and cause transcriptional pausing or termination, act as miR sponges and upregulate the miR target mRNAs, interact with RNA binding proteins (RBPs) to regulate target genes expression and so on. But in recent years, a few circRNAs have been identified to directly or indirectly recruit ribosomes and be translated [48]. According to our data, hsa_circ_0004085 enhanced the stability of GRP78 mRNA by binding to RRBP1, which has been confirmed to regulate the mRNA stability or enhance the binding of the mRNA to the ribosome [27]. In addition, hsa_circ_0004085 showed its influence on subcellular translocation of ATF6p50. Such function has been rarely studied in tumors. In cardiomyopathy, it has been reported that circ-Foxo3 interacts with anti-senescent proteins ID-1 and E2F1, as well as anti-stress proteins FAK and HIF1α, leading to the relocation of these proteins in cytoplasm [49]. The regulatory of circRNAs on target genes often exhibit as a network and we cannot rule out the diverse regulatory mechanisms of hsa_circ_0004085 on other genes. These unknowns and uncertainties inspire us to conduct deeper and broader research.

## Conclusion

**Fig. 9:**
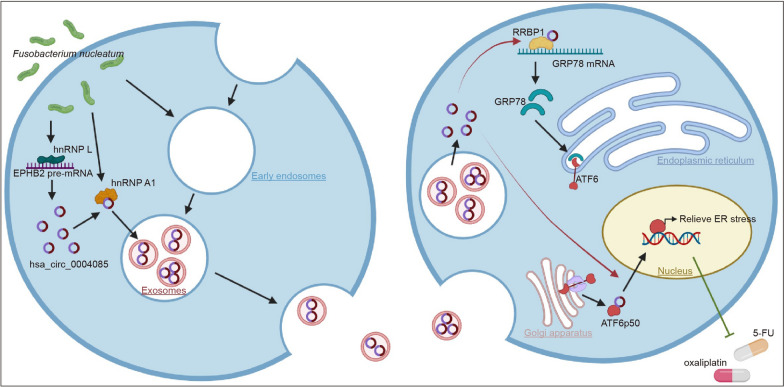
Fn infection promoted hsa_circ_0004085 formation by hnRNP L and packaged hsa_circ_0004085 into exosomes by hnRNP A1. Exosomes secreted by *Fusobacterium nucleatum*-infected colon cancer deliver hsa_circ_0004085 between cells. Hsa_circ_0004085 delivered in this way relieves ER stress in recipient cells by regulating GRP78 and ATF6p50, thereby delivering resistance to oxaliplatin and 5-FU

In summary, plasma levels of hsa_circ_0004085 are increased in colon cancer patients with intracellular Fn and are associated with a poor response to oxaliplatin/5-FU. Fn infection promoted hsa_circ_0004085 formation by hnRNP L and packaged hsa_circ_0004085 into exosomes by hnRNP A1. Cell-to-cell transfer of hsa_circ_0004085 mediated by exosomes produced by Fn-infected CRC cells spread oxaliplatin or 5-FU resistance by relieving ER stress in recipient cells. Mechanistically, hsa_circ_0004085 enhanced the stability of GRP78 mRNA by binding to RRBP1 and promoted the nuclear translocation of ATF6p50 to relieve ER stress (Fig. 9).

### Supplementary Information

**Additional file 1:** Supplementary Material.

**Additional file 2:** Sequence of primers/probes/siRNAs/shRNA.

**Additional file 3: **LC-MS/MS analysis of pulldown products.

**Additional file 4: **Source and catalog number of antibodies.

## Data Availability

The datasets used and/or analyzed during the current study are available upon reasonable request.
