# Supplementary material for: Exosomes secreted by Fusobacterium nucleatum-infected colon cancer cells transmit resistance to oxaliplatin and 5-FU by delivering hsa_circ_0004085
Source: J Nanobiotechnology. 2024 Feb 15;22:62. doi: 10.1186/s12951-024-02331-9 (PMC10867993; doi:10.1186/s12951-024-02331-9)
Supplement: Supplementary file 1 — Additional file 1: Supplementary Material. [file 12951_2024_2331_MOESM1_ESM.docx]

**Supplemental Methods**

CircRNA sequencing

Total RNA from each sample was extracted using TRIzol (Invitrogen, CA, USA) following the manufacturer's instructions. After quality inspection, Ribo-Zero^TM^ rRNA Removal Kit (Epicentre, Wisconsin, USA) was used to remove ribosomal RNA from total RNA, and linear RNA was digested using RNase R (Epicentre, Wisconsin, USA). RNA with PolyA tails was retrieved using magnetic beads with oligo-dT to maximize the elimination of linear RNA. The RNA was subsequently randomly interrupted by ion interruption using divalent cations. The synthetic cDNA was screened, amplified, purified and the library was obtained. The mixed libraries were gradually diluted and then sequenced in PE150 mode on a Illumina sequencer. After data quality control of the results, the sequence was aligned to the reference genome. We performed a bioinformatics analysis of differential circRNA after analyzing the expression level and expression differences.

Quantitative real time polymerase chain reaction (qRT-PCR) and quantitative polymerase chain reaction (qPCR)

Total RNA was extracted from a fixed volume of medium or plasma with mirVana PARIS Kit (Ambion, USA) or extracted from tissues or cells using TRIzol (Takara, Japan). Samples obtained after reverse transcription were tested by qRT-PCR at ABI 7900HT Fast Real-Time PCR System (Applied Biosystems, USA) using SYBR Green Master Mix (Takara, Shiga, Japan). RNA levels in cell or tissue lysates were normalized against GAPDH, while RNA levels in medium, plasma, and exosomes were normalized against the exogenous reference gene λpolyA. DNA was extracted from tissue samples using AllPrep DNA / RNA Mini Kit (Qiagen, Hilden, Germany) and tested by qPCR using the Fn primers. The Prostaglandin transporter (PGT) was used as an internal reference to calculate the Fn load in the tumor tissues. The primers are listed in Supplementary Table 1.

Culture of primary tumor cells

CRC tissues were collected aseptically and wetly from 4 CRC patients who underwent surgery before chemotherapy. Mechanical damage to the cells was minimized during the sampling process. Fresh tissues were immersed in culture medium and transported to the laboratory on ice within 15 minutes. CRC tissues were washed with Hanks solution three times and were cut into pieces about 4 mm in diameter. The tissue fragments were washed three times to remove blood cells and adipose tissue. 0.25% trypsin was added to the pieces and placed at 4℃ overnight. The next day, after washed with Hanks solution, tissue fragments are separated into single-cell primary cells with a small amount of culture medium. Primary CRC cells were cultured at the appropriate cell concentration. All of these CRC tissues were confirmed as icFn- by qRT-PCR for subsequently extract of primary CRC cells and for infection experiments of living Fn or heat-inactivated Fn.

Culture of colon cancer cell lines

Four colon cancer cell lines (LOVO, SW 480, HCT116, and DLD1) were selected for the study. Among them, LOVO and SW480 were purchased from China Center for Type Culture Collection, while HCT116 and DLD1 were obtained from the National Biomedical Cell-line Resource Center. They were grown in RPMI 1640 (GIBCO-BRL) or DMEM (GIBCO-BRL) medium containing 10% fetal bovine serum, 100 U/mL penicillin and 100 mg/mL streptomycin in a 37℃ incubator at 5% CO_2_.

Bioinformatics analysis

Circinteractome database (https://circinteractome.irp.nia.nih.gov/), NCBI database (https://www.ncbi.nlm.nih.gov/), and UCSC Genome Browser (http://genome.ucsc.edu/) were used to identify the origin of hsa_circ_0004085. The GEPIA2 Analysis Platform (http://gepia2.cancer-pku.cn/#index) was used to analyze the sequencing data of the Cancer Genome Atlas (TCGA) and Genotype-Tissue Expression (GTEx).

Sanger sequencing

The product of qRT-PCR was subjected to Sanger sequencing to detect the spliced region of hsa_circ_0004085, thus confirming its circular structure. Divergent and convergent primers were designed for RT-PCR to exclude head-to-tail splicing from trans-splicing or genomic rearrangements.

Actinomycin D and RNase R treatment

CRC cell lines were seeded in a 24-well plate and cultured overnight. Total RNAs were extracted from cells using TRIzol (Invitrogen, California, USA) and Isopropanol (Aladdin, Shanghai, China) after actinomycin D (2 mg/mL; MedChemExpress, Shanghai, China) or DMSO (Solarbio, Beijing, China) treatment for different periods. An equivalent of 5 μg RNA was incubated with or without RNase R (20 U; Thermo Fisher Scientific, Waltham, USA) at 37℃ for different periods. After treatment with actinomycin D or RNase R, the relative expression of hsa_circ_0004085 or EPHB2 mRNA was detected by qRT-PCR.

Collection of clinical specimens and clinicopathological information

We studied three cohorts of patients with CRC. CircRNA sequencing was performed in tissue samples in Cohort 1 containing 4 CRC^icFn+^ patients and 4 CRC^icFn-^ patients to determine the aberrantly-expressed circRNA in CRC infected with Fn. Expression levels of circRNA were examined in the tumor tissues or plasma of the 32 CRC patients and 32 healthy donors. CRC patients in cohort 2 had not received any treatment before surgery and they donated peripheral blood samples before, after surgery, and after tumor recurrence / metastasis. Cohort 3 contained 40 CRC patients who received chemotherapy regimen mainly based on XELOX or FOLFOX and they donated peripheral blood samples before chemotherapy. CRC tissues was frozen in liquid nitrogen or embedded in paraffin until use, while blood samples were centrifuged at 2,500 g for 10 min to extract serum and then stored at -80℃ until use. The disease progression was monitored by Medical Image System, gastrointestinal endoscopy and telephone follow-up. Patients were divided into two groups according to RECIST 1.1 criteria. Non-progressive patients included "complete response", "partial response" and "stable disease", while resistant patients included "disease progression".

Western blot assay

Total protein was extracted using RIPA Lysis buffer containing PMSF and cocktail, while nuclear and cytoplasmic proteins were extracted respectively using NE-PER ™ Nuclear and Cytoplasmic Extraction kit (78833, Thermo Scientific). Protein concentration was determined with BCA Protein Assay kit. After gel electrophoresis, the proteins were transferred from the gel to a methanol-activated PVDF membrane. The membranes were blocked with 5% milk and probed with the primary antibodies at 4◦C overnight, followed by incubation with the secondary antibodies at room temperature for 1 hour. The membrane was incubated with 0.125 ml/cm^2^ High sensitive ECL luminescence reagent for 2 min before detected and analyzed with Automatic Chemiluminescence Image Analysis System. The antibodies are listed in Supplementary Table 3.

Cell proliferation assay

For CCK-8 assay, CRC cells were seeded in a 96-well plate and cultured with medium containing Oxa or 5-Fu. After that, each well was supplemented with 10 µL of CCK-8 reagent (Dojindo, Kumamoto, Japan) and incubated for 3 h at 37 °C. The absorbance was measured by using a microplate reader (Bio-Rad, USA). For colony formation assay, CRC cells were plated in a six-well plate. Cells were treated with Oxa or 5-Fu for 48 h and then cultured with normal medium for 3 weeks. 0.1% crystal violet was used to stain the cells and the colonies were counted. For 5-ethynyl2′deoxyuridine (EdU) assay, CRC cells were seeded in a 48-well plate and cultured with medium containing Oxa or 5-Fu. After 48 h, each well was supplemented with EdU reagents (Solarbio, Beijing, China) according to the manufacturer’s instructions. Fluorescence microscopy (Olympus, Tokyo, Japan) was used to capture the images. For colony formation assay, 500 cells were plated in a six-well plate and cultured for 3 weeks. Then, 0.1% crystal violet was used to stain the cells and the colonies were counted.

Animal study

5×10^6^ CRC cells were injected subcutaneously into 4-week-old male BALB/C nude mice (Vitonlihua, Beijing, China) to construct a xenograft model of nude mice. One week later, Oxa (7.5 mg/kg) was injected intraperitoneally every three days while 5-FU (23 mg/kg) was injected intraperitoneally every two days. Mice were sacrificed on day 20 for further analysis, during which the tumor size and the body weight of the mice were measured every five days. The tumor volume (V) was calculated by the following formula: V= (ab^2^) / 2, where a and b represent the longer and shorter tumor diameters respectively.

Apoptosis Detection

Apoptosis was examined by flow cytometric analysis. An Annexin V FITC/PI double stain assay (BD Biosciences, San Jose, CA) was performed following the manufacturer’s protocol.

Immunofluorescence (IF)

Cells were fixed with 4% paraformaldehyde at room temperature, washed three times and then permeabilized with 0.5% Triton-X100 at room temperature. After blocked, the cells were incubated with antibodies specific for PDI, RRBP1 or ATF6 at 4◦C overnight, and then with correspondent secondary antibodies at 37◦C for 2 h. Subsequently, the slides were washed with PBS. Finally, the nuclei were stained with DAPI (Thermo Fisher Scientific, Waltham, USA), and the IF signal was visualized under a confocal microscopy (ZEISS, LSM880, Oberkochen, Germany). The antibodies are listed in Supplementary Table 3.

Immunohistochemistry (IHC)

For IHC, the paraffin sections were deparaffinized and dehydrated by xylene and a series of graded ethanol. Paraffin-embedded tissues were subject to IHC analysis to determine the protein expression. After incubated with primary antibodies at 4◦C overnight, the tissues were incubated with secondary antibody for 1 h at room temperature. Histochemistry score (H-SCORE) were analyzed using the Aipathwell Digital Pathology Image Analysis Software (Servicebio). The antibodies are listed in Supplementary Table 3.

RNA pulldown assay

RNA pull-down experiments were performed using the Pierce™ magnetic RNA-Protein Pull-Down kit (Cat. 20164, Thermo). According to the manufacturer's instructions, the probes were added to the lysate and rotated for 16–24 h at room temperature. Then, 100 µl streptavidin magnetic beads were added to the lysis solution and rotated at room temperature for 2–4 h. Subsequently, a magnetic stand was used to collect the magnetic beads, which were then washed five times with washing buffer (containing PMSF, Protease inhibitor, and RNase inhibitor). Then, the magnetic beads were resuspended in 1 ml washing buffer (containing PMSF, Protease inhibitor, and RNase inhibitor), of which 100 µl was collected for RNA purification and 900 µl was collected for protein purification. For RNA extraction, the 100 µl sample was mixed with 5 µl proteinase K (Sangon Biotech, Shanghai, China) and RNA PK buffer, followed by mild rotating at 50 °C for 45 min, then 95 °C for 10 min to break the formaldehyde cross-links. Next, the RNA was purified by TRIzol reagent (Invitrogen, Carlsbad, CA, USA), and the RNA was reverse transcribed to cDNA and stored at −80℃ for further use. For protein extraction, 300 µl 4×loading buffer was added to the remaining 900 µl sample and incubated at 100 °C for 10 min. Next, the supernatant containing the protein product was separated by the magnetic stand, and the protein was further used for mass spectrometry (MS) analysis and western blot. The sequences of probes are listed in Supplementary Table 1.

RNA immunoprecipitation (RIP) assay

The RIP assay was performed using Magna RIP™ RNA binding protein immunoprecipitation kit (Cat.17-701, Millipore, USA). Briefly, CRC cells were collected and lysed by RIP lysis buffer. The lysis products were added to RIP reaction buffer containing magnetic beads-antibody complexes and incubated overnight. Then, the coprecipitated RNAs were purified by TRIzol reagent (Invitrogen, Carlsbad, CA, USA) and analyzed by qRT–PCR. The antibodies are listed in Supplementary Table 3.

**Supplemental Figures and legends:**

**
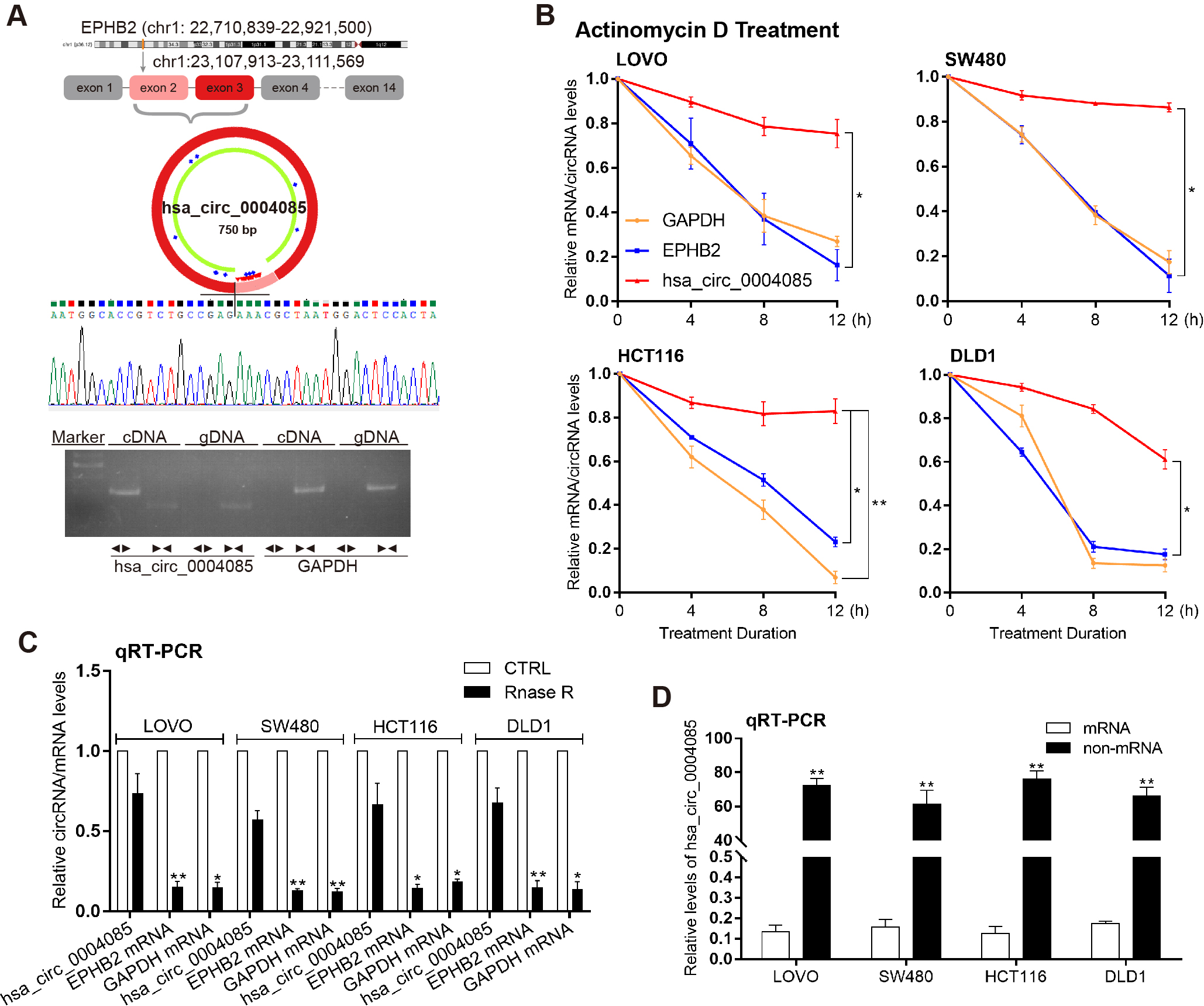
**

**Figure S1. Identification of the origin and cyclic structure of hsa_circ_0004085.** (A) Origin of hsa_circ_0004085 was identified Circinteractome database, NCBI database and UCSC Genome Browser (top). Back Splice Point sequence of hsa_circ_0004085 was determined by Sanger sequencing (middle). Divergent and convergent primers were designed for RT-PCR (lower). (B) The stability of hsa_circ_0004085, GAPDH mRNA, and EPHB2 mRNA was examined by treating the cells with actinomycin D. (C) Degradation of hsa_circ_0004085, GAPDH mRNA, and EPHB2 mRNA was examined after treating the total RNA with RNase R. (D) The expression level of hsa_circ_0004085 was determined after the purification of Poly-A RNA. (*P<0.05, **P<0.01, ***P<0.001, NS: not signifcant)


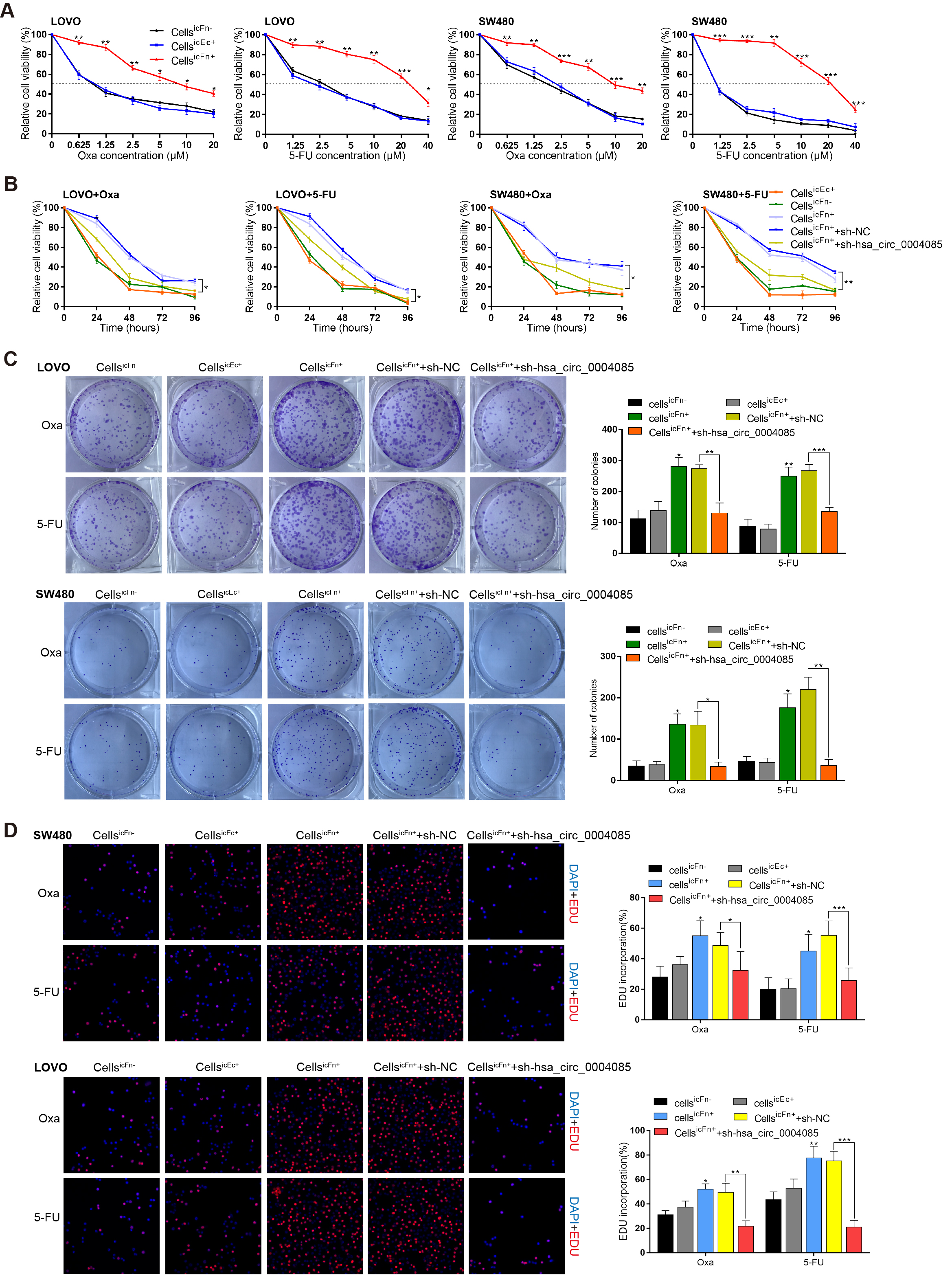


**Figure S2. Fn-infection conferred CRC cells resistance to oxaliplatin/5-FU by hsa_circ_0004085.** (A) CCK-8 analysis detected the survival of Cells^icFn+^, Cells^icFn-^, and Cells^icEc+^ in different concentrations of Oxa/5-Fu and to obtain IC50 of Oxa/5-Fu for CRC cells. (B-D) Resistance to Oxa/5-Fu of Cells^icFn+^, Cells^icFn-^, and Cells^icEc+^ was analyzed by CCK-8 analysis, colony formation and EDU experiments. (*P<0.05, **P<0.01, ***P<0.001, NS: not signifcant)

**
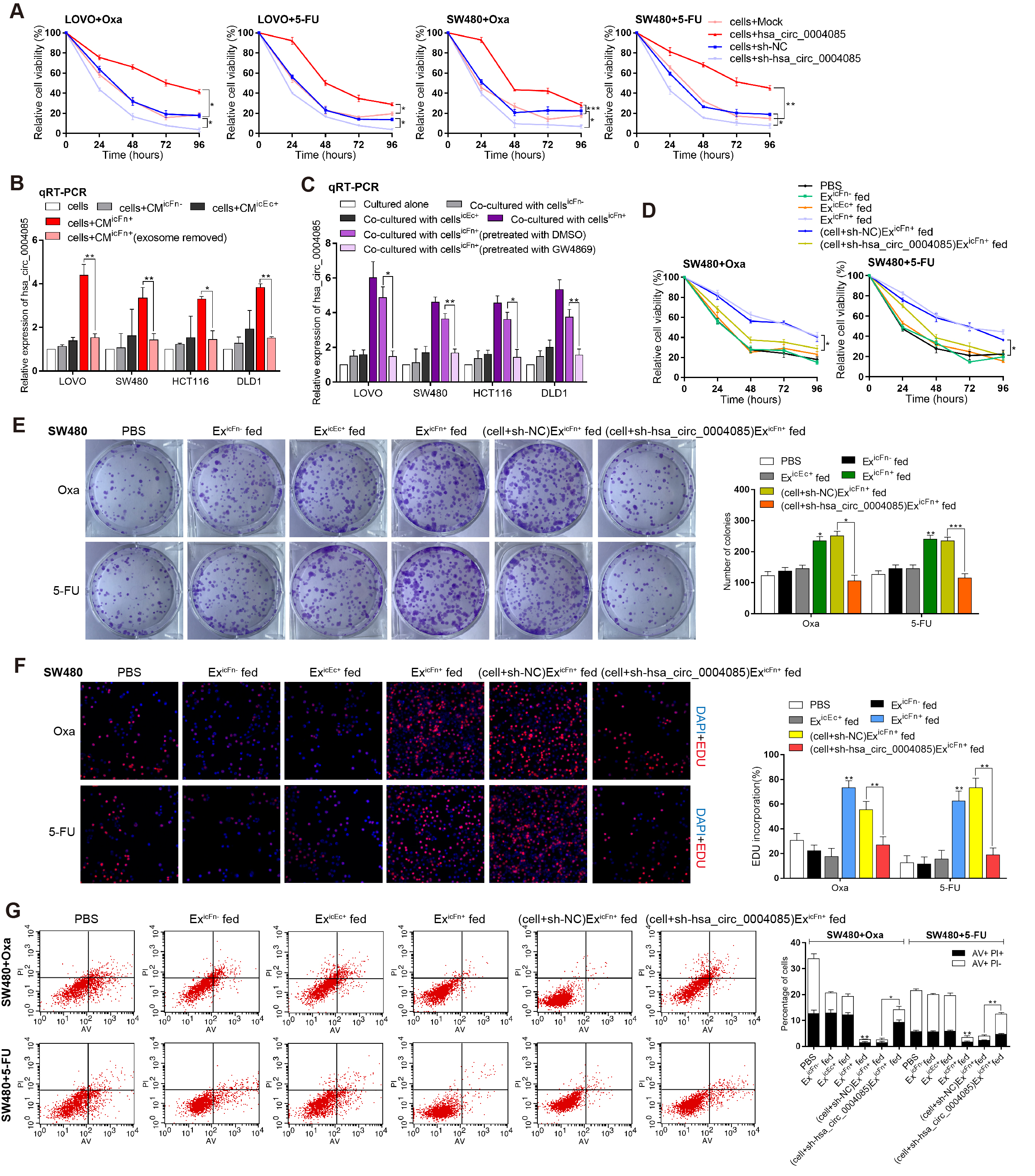
**

**Figure S3.** **Ex^icFn+^ tansmitted resistance to oxaliplatin/5-FU by delivering hsa_circ_0004085 in vitro.** (A) Resistance to Oxa/5-Fu in cells with high or low levels of hsa_circ_0004085 was analyzed by CCK-8 analysis. (B-C) The levels of hsa_circ_0004085 in CRC cells incubated with CM^icFn+^, CM^icFn-^ or CM^icEc+^ were determined by qRT-PCR. (D-F) Resistance to Oxa/5-Fu of SW480 cells incubated directly with Ex^icFn+^, Ex^icFn-^ or Ex^icEc+^ was analyzed by CCK-8 analysis, colony formation and EDU experiments. (G) Oxa/5-Fu-induced apoptosis of SW480 cells incubated directly with Ex^icFn+^, Ex^icFn-^ or Ex^icEc+^ was tested with flow cytometry. (*P<0.05, **P<0.01, ***P<0.001, NS: not signifcant)

**
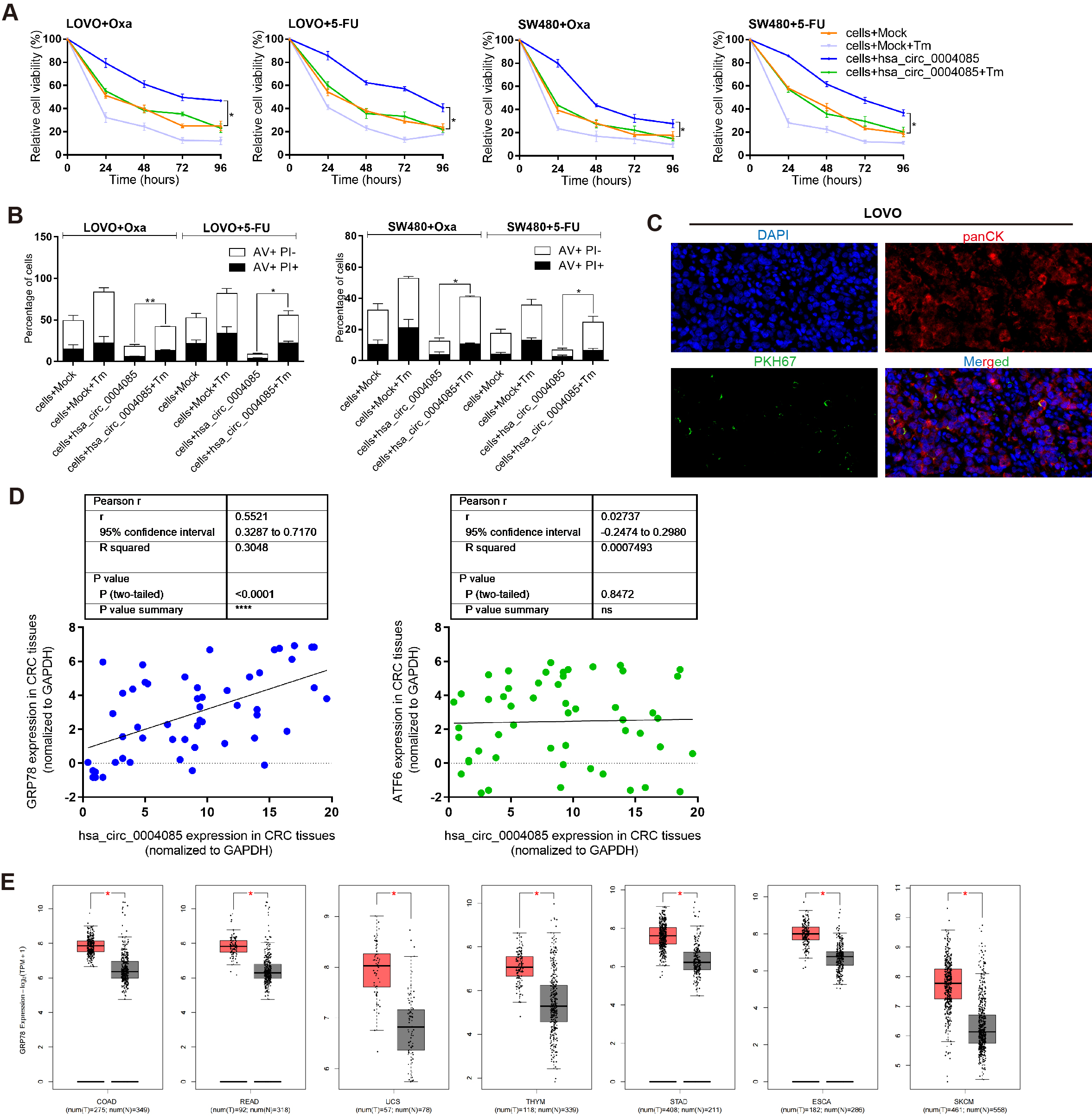
**

**Figure S4. Tm partly abolished the resistance to oxaliplatin/5-FU induced by hsa_circ_0004085.** (A) CCK-8 analysis examined the influence of hsa_circ_0004085 on resistance of CRC cells to Oxa/5-Fu in the presence of Tm. (B) Flow cytometry tested the influence of hsa_circ_0004085 on Oxa/5-Fu-induced apoptosis of CRC cells in the presence of Tm. (C) Exosomes containing hsa_circ_0004085 were extracted, labeled with PKH67 (green), and injected into xenograft formed by LOVO cells. Transplanted tumors were harvested 24 hours later for IF experiments. DAPI (blue): cell nucleus, panCK (red): epithelial tumor. (D) The correlation of hsa_circ_0004085 with GRP78 mRNA or ATF6 mRNA levels was analyzed in CRC tumor tissues. (E) GRP78 expression levels in multiple tumors were analyzed using GEPIA to resolve the sequencing data derived from TCGA and GTEx. (*P<0.05, **P<0.01, ***P<0.001, NS: not signifcant)

**
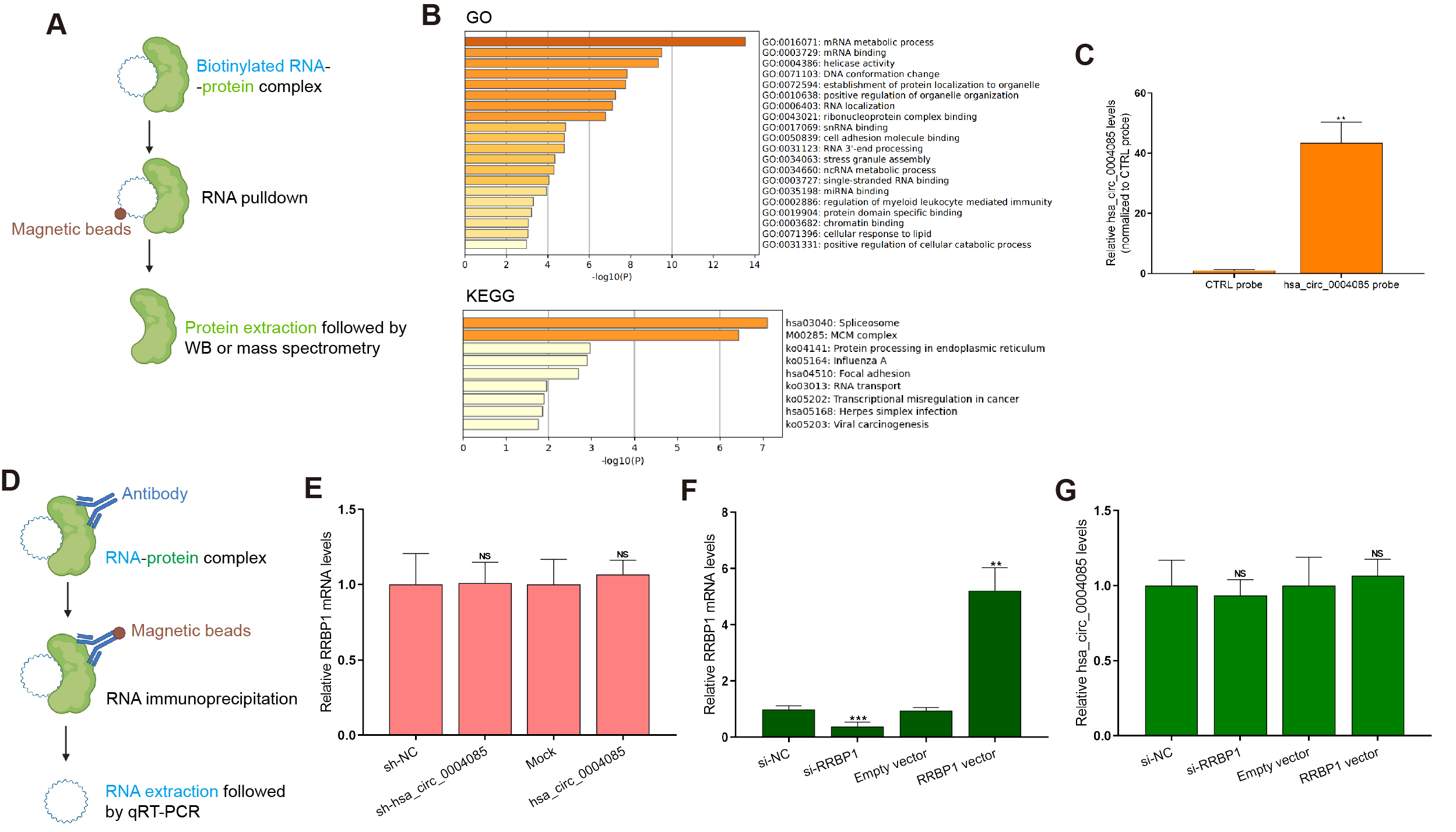
**

**Figure S5. Hsa_circ_0004085 functioned by binding RRBP1.** (A) Pulldown experiments were performed using probes designed according to the junction region of hsa_circ_0004085. (B) GO analysis revealed that the proteins specifically bound by hsa_circ_0004085 were associated with various functions. KEGG database showed that the proteins specifically bound by hsa_circ_0004085 were located in multiple signaling pathways. (C) The pull-down efficiency of hsa_circ_0004085 probe was tested by qRT-PCR. (D) RIP experiments were performed using anti-RRBP 1 antibody or anti-IgG antibody. (E) QRT-PCR detected the influence of hsa_circ_0004085 on RRBP1 mRNA. (F-G) QRT-PCR detected the expression levels of hsa_circ_0004085 and RRBP1 mRNA after knockdown or overexpression of RRBP1. (*P<0.05, **P<0.01, ***P<0.001, NS: not signifcant)


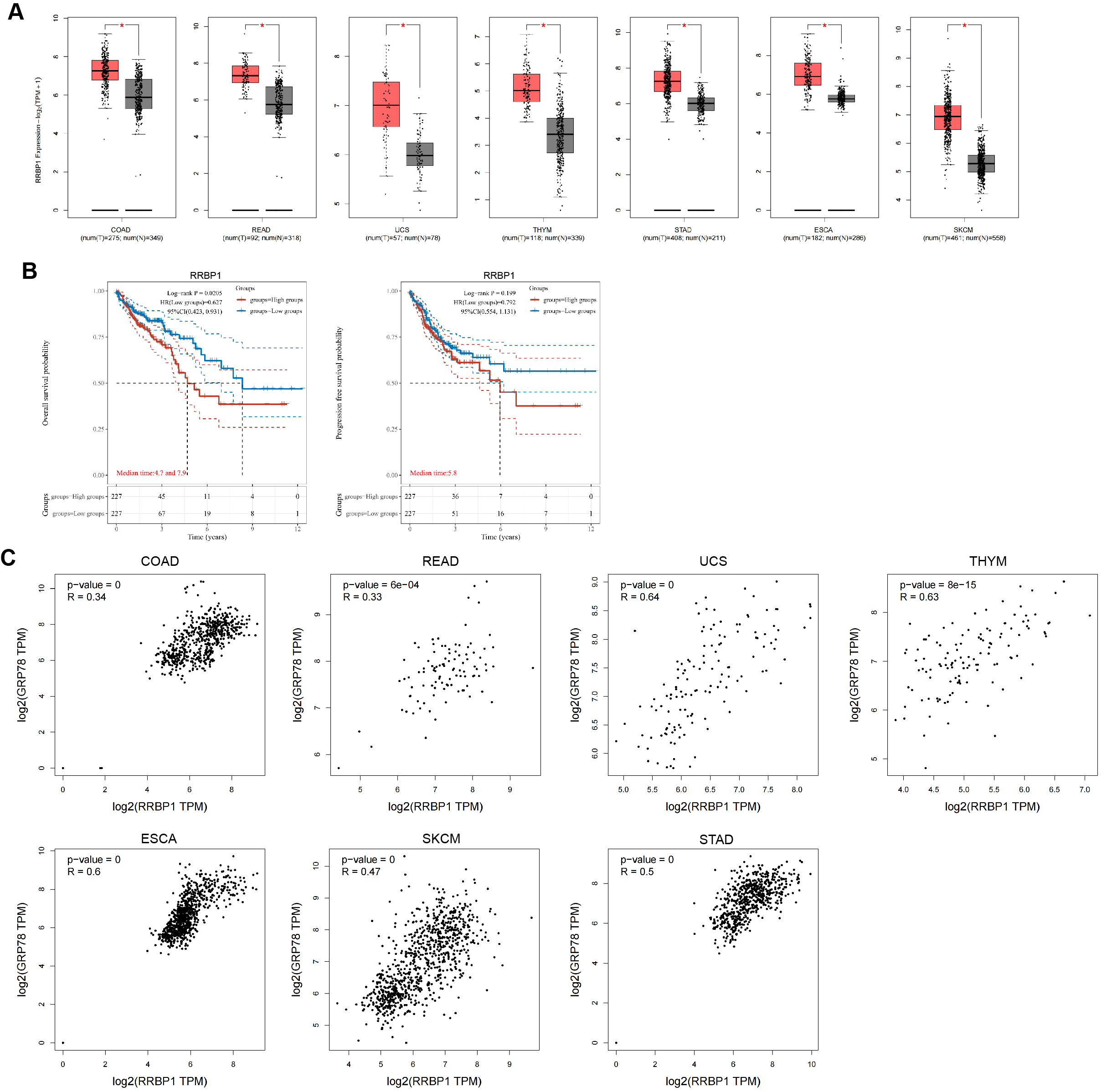


**Figure S6. Bioinformatics analysis of RRBP1 and GRP78.** (A) GEPIA analyzed expression levels of RRBP1 in multiple tumors in the TCGA and GTEx databases. (B) GEPIA survival analysis of OS (left) and PFS (right) in patients with high or low level of RRBP1. (C) GEPIA analysis of the correlation between RRBP1 and GRP78 expression levels. (*P<0.05, **P<0.01, ***P<0.001, NS: not signifcant)

**
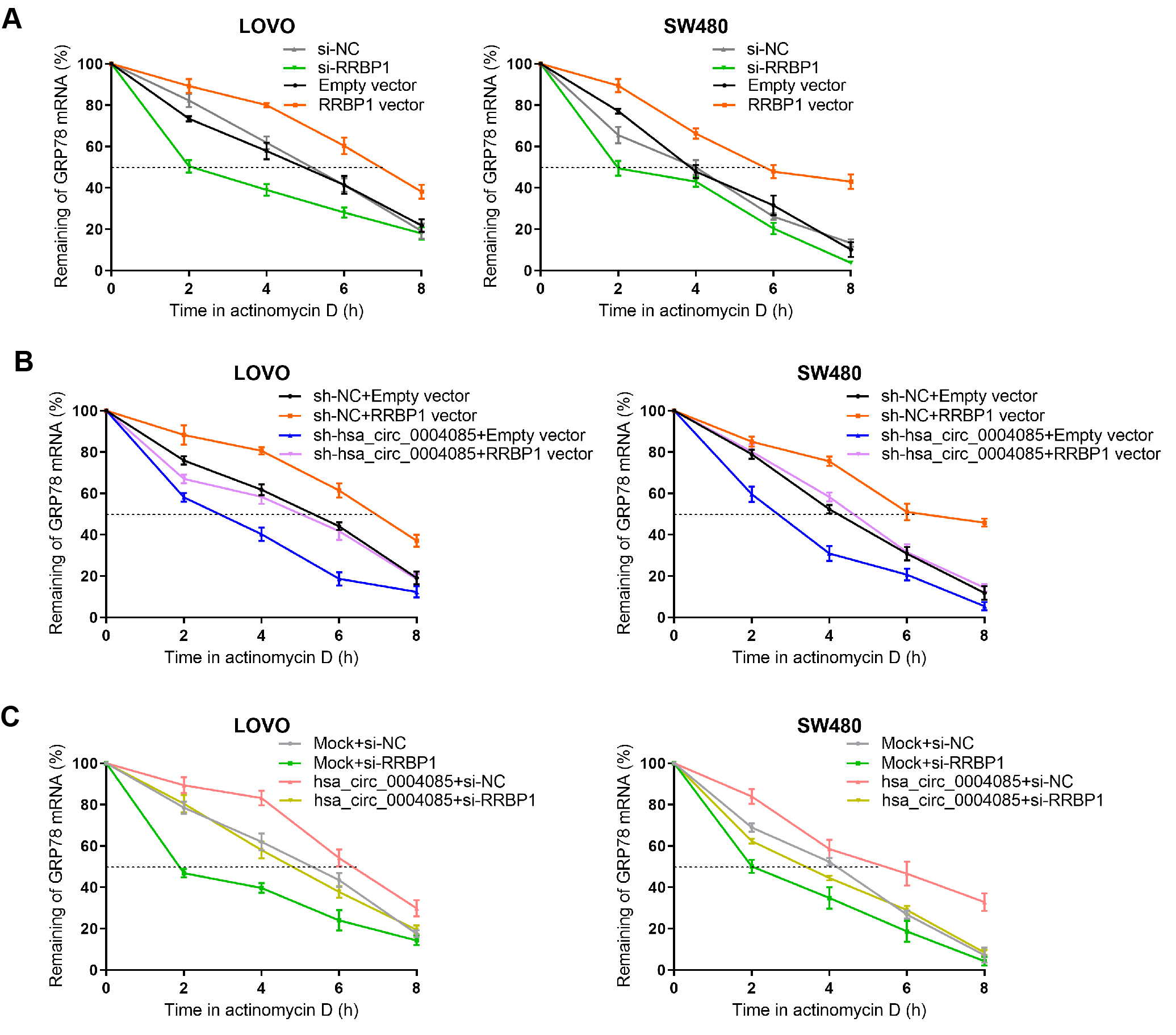
**

**Figure S7. Hsa_circ_0004085 enhances the stability of GRP78 mRNA by regulating RRBP1.** (A) The effect of RRBP1 on stability of GRP78 mRNA was tested in CRC cells treated with actinomycin D. (B) The effect of sh-hsa_circ_0004085 and RRBP1 vector on half-life of GRP78 mRNA was tested in CRC cells treated with actinomycin D. (C) The effect of hsa_circ_0004085 vector and si-RRBP1 vector on half-life of GRP78 mRNA was tested in CRC cells treated with actinomycin D. (*P<0.05, **P<0.01, ***P<0.001, NS: not signifcant)
